# Addition of m^6^A to SV40 late mRNAs enhances viral structural gene expression and replication

**DOI:** 10.1371/journal.ppat.1006919

**Published:** 2018-02-15

**Authors:** Kevin Tsai, David G. Courtney, Bryan R. Cullen

**Affiliations:** Department of Molecular Genetics and Microbiology, Duke University Medical Center, Durham, North Carolina, United States of America; Tulane Health Sciences Center, UNITED STATES

## Abstract

Polyomaviruses are a family of small DNA tumor viruses that includes several pathogenic human members, including Merkel cell polyomavirus, BK virus and JC virus. As is characteristic of DNA tumor viruses, gene expression in polyomaviruses is temporally regulated into an early phase, consisting of the viral regulatory proteins, and a late phase, consisting of the viral structural proteins. Previously, the late transcripts expressed by the prototypic polyomavirus simian virus 40 (SV40) were reported to contain several adenosines bearing methyl groups at the N^6^ position (m^6^A), although the precise location of these m^6^A residues, and their phenotypic effects, have not been investigated. Here, we first demonstrate that overexpression of the key m^6^A reader protein YTHDF2 induces more rapid viral replication, and larger viral plaques, in SV40 infected BSC40 cells, while mutational inactivation of the endogenous *YTHDF2* gene, or the m^6^A methyltransferase METTL3, has the opposite effect, thus suggesting a positive role for m^6^A in the regulation of SV40 gene expression. To directly test this hypothesis, we mapped sites of m^6^A addition on SV40 transcripts and identified two m^6^A sites on the viral early transcripts and eleven m^6^A sites on the late mRNAs. Using synonymous mutations, we inactivated the majority of the m^6^A sites on the SV40 late mRNAs and observed that the resultant viral mutant replicated more slowly than wild type SV40. Alternative splicing of SV40 late mRNAs was unaffected by the reduction in m^6^A residues and our data instead suggest that m^6^A enhances the translation of viral late transcripts. Together, these data argue that the addition of m^6^A residues to the late transcripts encoded by SV40 plays an important role in enhancing viral gene expression and, hence, replication.

## Author summary

All organisms, including all viruses, use mRNAs to express the proteins required for their viability and replication. Viruses, in particular, are under pressure to replicate rapidly in order to spread before the host immune response becomes effective. Previously, it was reported that several viruses, including polyomaviruses, express mRNAs that bear methylated adenosine (m^6^A) residues, but the functional relevance of this modification was unclear. Here, we demonstrate that addition of m^6^A to mRNAs encoded by the prototypic polyomavirus SV40 increases both viral structural protein expression and viral spread. Targeting the sites of addition of m^6^A on SV40 mRNAs, using mutagenesis, therefore reduces SV40 replication, and a drug that globally inhibits m^6^A addition has a similar effect. These data identify m^6^A addition as a key step, and potential Achilles heel, in the polyomavirus life cycle.

## Introduction

Mammalian mRNAs are subject to a number of covalent modifications at the single nucleotide level, of which addition of a methyl group to the N^6^ position of adenosine (m^6^A) is the most prevalent [1,2]. m^6^A has been reported to be deposited co-transcriptionally by a heterotrimeric “writer” complex consisting of the enzyme methyltransferase like 3 (METTL3) and two co-factors called METTL14 and WTAP. m^6^A residues are detected by “reader” proteins containing a YTH domain that binds m^6^A directly [1–4]. Human cells express two predominantly nuclear m^6^A readers, called YTH Domain-containing 1 (YTHDC1) and 2, which have been proposed to regulate the splicing of m^6^A-containing RNAs and to facilitate their nuclear export [5–8]. Once in the cytoplasm, m^6^A residues are bound by three additional readers, called YTH Domain Family 1 (YTHDF1), 2 and 3, which have been proposed to regulate the translation and stability of m^6^A-containing mRNAs [1,2].

While m^6^A is essential for the development of multicellular organisms [9,10], and plays a key role in regulating several aspects of cellular mRNA function [1,2], the role of m^6^A in regulating viral gene expression has been less clear [11,12]. However, it has been known for almost 40 years that a wide variety of viral mRNAs are heavily m^6^A modified, which suggests that m^6^A is likely facilitating some aspect(s) of viral mRNA function. Consistent with this hypothesis, we have previously reported that m^6^A sites present on transcripts encoded by the pathogenic human viruses human immunodeficiency virus 1 (HIV-1) and influenza A virus (IAV) significantly enhance viral mRNA and protein expression in *cis* and, in the case of IAV, also increase viral pathogenicity [13,14]. Moreover, we have also reported that the mutational inactivation of the METTL3 “writer” or the YTHDF2 “reader” protein strongly inhibits IAV and HIV-1 replication, respectively, while overexpression of the key m^6^A reader protein YTHDF2 strongly enhances both HIV-1 and IAV replication in culture [13,14]. While others have confirmed that m^6^A addition is important for HIV-1 replication in culture [15], it has also been reported that m^6^A attenuates the replication of hepatitis C virus (HCV) and the distantly related flavivirus Zika virus (ZKV) [16,17], though why an acute virus such as ZKV should retain m^6^A sites if they inhibit viral gene expression in *cis* is currently unclear.

As noted above, m^6^A has been detected on transcripts encoded by a wide variety of viruses, including not only RNA viruses but also DNA tumor viruses, including the simian polyomavirus SV40 [13,18]. In particular, SV40 was reported in 1979 to contain several m^6^A residues in transcripts derived from the viral “late” region, all of which were in the consensus sequence 5’-RAC-3’, where R is purine, although “G” at position one was more prevalent than “A” [19]. However, the precise location of these m^6^A residues was not determined and their functional significance has remained unclear. Here, we first demonstrate that the replication of SV40, like the replication of HIV-1 and IAV, is substantially enhanced by overexpression of the key m^6^A reader protein YTHDF2, while mutational inactivation of either the *YTHDF2* or *METTL3* gene in the permissive cell line BSC40 inhibited SV40 replication. Using two distinct techniques, photo-crosslinking-assisted m^6^A sequencing (PA-m^6^A-seq) [20] and photoactivatable ribonucleoside-enhanced crosslinking and immunoprecipitation (PAR-CLIP) [21] of epitope-tagged YTHDF proteins, we next identified and precisely mapped 11 m^6^A peaks on the SV40 late transcripts, and two peaks in the SV40 early region. We then used silent mutagenesis to specifically ablate several SV40 late region m^6^A addition sites and we observed that this markedly inhibits SV40 replication, as well as SV40 structural gene expression, but does not affect the pattern of late gene mRNA splicing. Together, these data argue that m^6^A addition serves to increase the replication potential of the polyomavirus SV40.

## Results

### YTHDF2 enhances SV40 replication in culture

**Fig 1:**
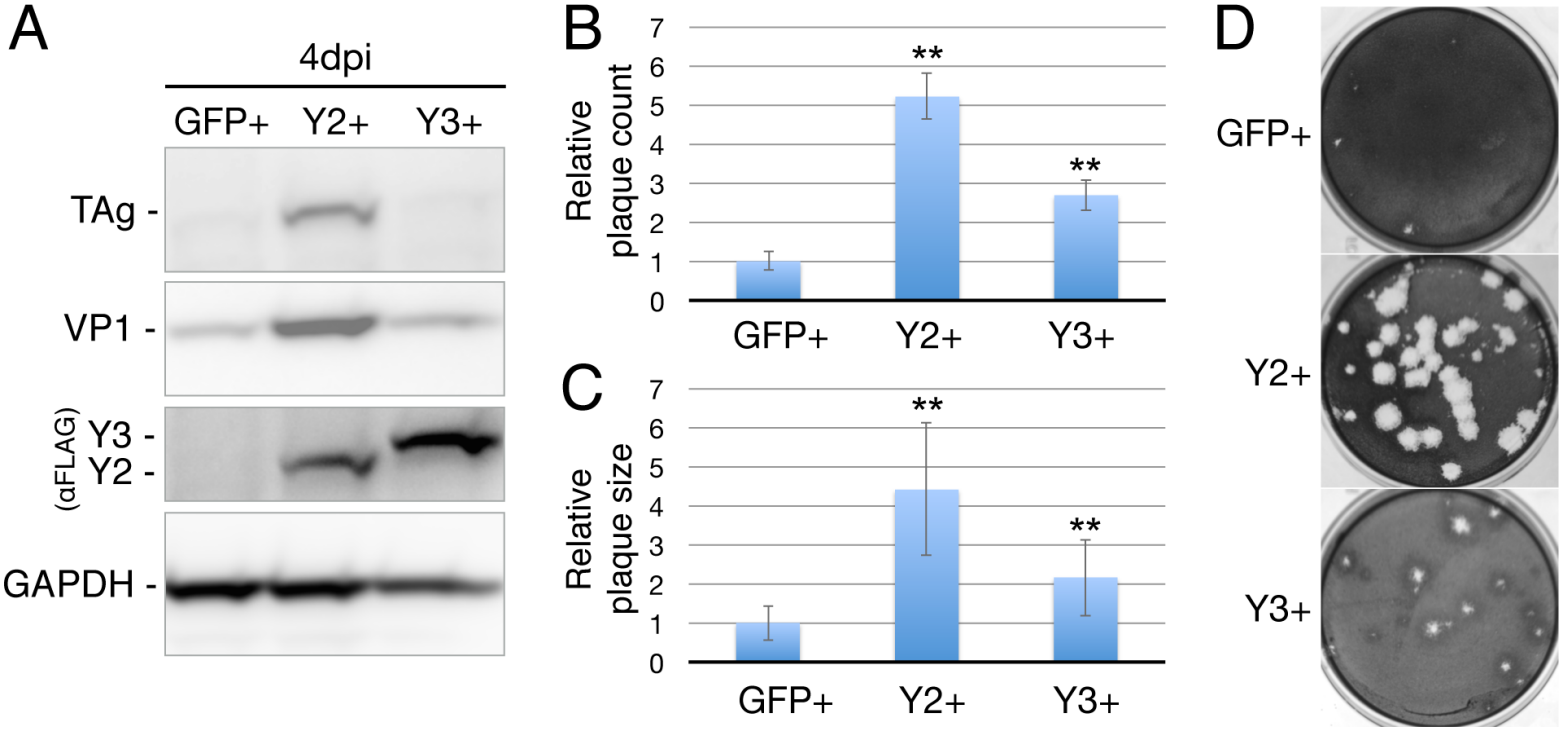
YTHDF2 overexpression increases SV40 replication. Cellular clones expressing FLAG-tagged GFP, YTHDF2 (Y2), or YTHDF3 (Y3) were obtained by lentiviral transduction of BSC40 cells. (A) SV40 protein expression in infected cells was quantified by Western blot for the SV40 early protein large T antigen (TAg) and the SV40 structural protein VP1. YTHDF protein expression was visualized using a FLAG epitope specific antibody and endogenous GAPDH served as a loading control. Cells were infected at an MOI of 0.003 and collected and lysed 4 days post-infection (dpi). (B and C) Susceptibility to SV40 infection was tested by plaque assay on GFP, Y2 or Y3 overexpressing BSC40 cells. (B) Plaque counts and (C) plaque sizes (measured as diameter, n = 14) were quantified from four separate infections. Data were normalized to the average quantity and size of SV40 plaques detected in the control, GFP-expressing BSC40 cells, which was set at 1.0. Error bars denote SD. **p<0.01 by 2-tailed Student's T-test. (D) Representative photograph of SV40 plaques on GFP, Y2 or Y3 overexpressing BSC40 cells infected with the same dilution of an SV40 stock.

As noted above, we have previously reported that overexpression of the key cytoplasmic m^6^A reader protein YTHDF2 significantly enhances the replication of HIV-1 in CD4+ T cells, and of IAV in lung epithelial cells [13,14]. We therefore hypothesized that this phenotype might be a shared characteristic of viruses that exploit m^6^A to boost their replication potential. To test this hypothesis, we used lentiviral transduction to derive clonal cell lines, in the context of the SV40-permissive cell line BSC40, that overexpress FLAG-tagged versions of YTHDF2 or YTHDF3, or FLAG-GFP as a control (Fig 1A). Based on Western blot analysis (S1A Fig), we estimate that the overexpression of YTHDF2 in these cells is ~3-fold. Of note, although we actually overexpressed human YTHDF2 or YTHDF3 in the BSC40 cells, which are of African green monkey (AGM) origin, the sequence of YTHDF2 and YTHDF3 is completely conserved between humans and AGMs (S1B and S1C Fig) and this distinction is therefore moot. Next we infected each clonal cell line with wild type SV40 at an MOI of 0.003 and allowed viral spread to occur. As shown by Western blot for the viral regulatory protein large T antigen (TAg), and the viral structural protein VP1, YTHDF2 overexpression indeed induced a marked increase in the level of viral spread by 4 days post-infection (dpi), while YTHDF3 overexpression had little or no effect (Fig 1A).

Previously, we have reported that YTHDF2 overexpression not only enhances IAV replication in A549 cells but also significantly increases the size of plaques caused by IAV on this cell line [14]. To determine if this is also true for SV40 plaques on BSC40 cells, we infected BSC40 cells expressing FLAG-GFP, FLAG-YTHDF2 or FLAG-YTHDF3 with several dilutions of our SV40 viral stock and then analyzed SV40 plaque formation 8 days later. As expected, we were indeed able to observe a dramatic, ~4-fold increase in the diameter, and ~16-fold increase in the area, of SV40 plaques on the BSC40/YTHDF2 cells when compared to the BSC40/GFP control cell line (Fig 1C and 1D). In contrast, YTHDF3 increased SV40 plaque diameter by a more modest ~2-fold. Surprisingly, we also observed ~5-fold more SV40 plaques on the BSC40/YTHDF2 cell line, when compared to the BSC40/GFP control, while the BSC40/YTHDF3 cells were again intermediate in phenotype (Fig 1B).

**Fig 2:**
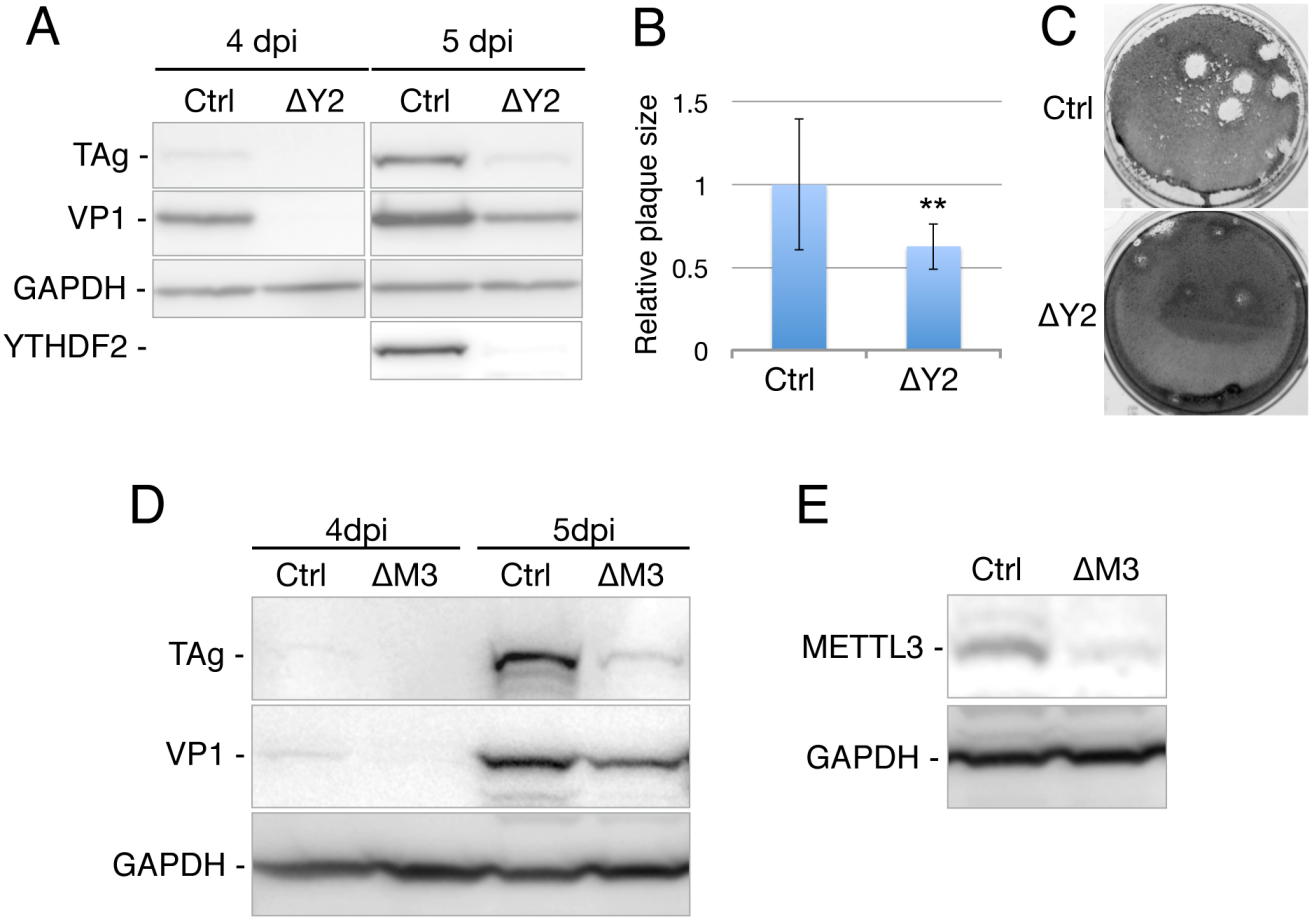
Knockout of YTHDF2 expression attenuates SV40 replication. (A) BSC40 cells were transduced with a lentiviral vector expressing Cas9 and an sgRNA targeting the YTHDF2 gene or a control sgRNA targeting GFP. A cell clone lacking YTHDF2 expression (ΔY2 cells) was then isolated. The control and ΔY2 cells were infected with SV40 at an MOI of 0.005 and viral protein and YTHDF2 expression probed by Western blot. (B) Plaque assays were performed on control or ΔY2 BSC40 cells using identical dilutions of an SV40 viral stock and plaque sizes (diameter) then determined. Average plaque size observed in control BSC40 cells was set at 1.0. SD as indicated, n = 12. **p<0.01 by 2-tailed T-test. (C) Representative photograph visualizing SV40 plaques on the control and ΔY2 cells. (D) Similar to panel A except that control (Ctrl) and METTL3 knockout BSC40 cells (ΔM3) were infected with SV40 at an MOI of 0.003 and viral protein expression then probed by Western blot at 4 and 5 dpi. (E) BSC40 cells were transduced with a lentiviral vector expressing Cas9 and an sgRNA targeting the METTL3 gene (ΔM3 cells) or a control sgRNA targeting GFP. Western blot was used to demonstrate that the ΔM3 cells in panel D indeed express a reduced level of METTL3.

If YTHDF2 overexpression enhances SV40 replication then one would predict that loss of the endogenous m^6^A reader YTHDF2 or m^6^A writer METTL3 would reduce SV40 replication, as we have indeed previously reported for HIV-1 and IAV [13,14]. To test this hypothesis, we used gene editing with CRISPR/Cas to inactivate the *YTHDF2* gene in a single cell clone of BSC40, called ΔY2. DNA sequencing of 16 independent PCR amplicons covering the targeted region of the *YTHDF2* gene confirmed that both alleles were indeed mutationally inactivated in the ΔY2 cells, with one allele showing a 41 base pair (bp) deletion at the predicted editing site while the second allele showed a 143 bp deletion (S2 Fig). Both of these unexpectedly large deletions are predicted to cause frame shift mutations in the YTHDF2 open reading frame (ORF). As confirmed by Western blot, the ΔY2 subclone did not express a detectable level of YTHDF2, even though YTHDF2 expression is readily detectable in wild type BSC40 cells (Fig 2A).

We next infected the ΔY2 cells, and control BSC40 cells, with SV40 at an MOI of 0.005 and then analyzed viral protein expression by Western blot at 4 dpi and 5 dpi (Fig 2A). As may be readily observed, the ΔY2 cells replicated SV40 significantly less effectively than control BSC40 cells, which express Cas9 and a single guide RNA (sgRNA) specific for the *gfp* gene. As expected, we also observed that SV40 plaques generated on the ΔY2 cells at 10 days post-infection were significantly smaller than those observed on the control BSC40 cells (Fig 2B and 2C).

If the inhibition of SV40 replication in the ΔY2 cells indeed results from the loss of YTHDF2 binding to viral m^6^A sites, then loss of the METTL3 m^6^A writer would be predicted to cause the same inhibitory effect. We therefore used CRISPR/Cas to also generate BSC40 cells in which the METTL3 gene had been mutationally inactivated, as confirmed by Western blot analysis (Fig 2E). As expected, the loss of METTL3 expression indeed resulted in a marked decrease in the spread of SV40 when compared to control BSC40 cells (Fig 2D).

### Mapping and mutational inactivation of SV40 late region m^6^A sites

As noted above, the SV40 late region has previously been reported to contain several sites of m^6^A modification [19], and the data presented in Figs 1 and 2 are consistent with the hypothesis that these m^6^A residues act to promote SV40 late gene expression and, hence, replication. However, it could also be argued that the observed positive effect of YTHDF2 on SV40 replication is indirect and due to effects on cellular, rather than viral, gene expression.

To address this concern, we used two previously published techniques, PA-m^6^A-seq and PAR-CLIP [20,21], to independently fine map m^6^A residues present on SV40 late mRNAs. Both PA-m^6^A-seq and PAR-CLIP involve the incubation of cells in the presence of the highly photoactivatable uridine analog 4-thiouridine (4SU), but while PA-m^6^A-seq uses UV irradiation to induce crosslinking of an m^6^A-specific antibody to poly(A)+ RNA isolated from the 4SU-treated cells, PAR-CLIP involves UV crosslinking of an RNA binding protein, in this case the FLAG-tagged m^6^A reader proteins YTHDF2 and YTHDF3, to bound m^6^A sites on mRNA in the living cell, followed by isolation of the resultant crosslinked ribonucleoprotein complex by IP for FLAG. Both techniques then subject the crosslinked complex to RNase treatment followed by isolation and deep sequencing of any protected RNA fragments.

**Fig 3:**
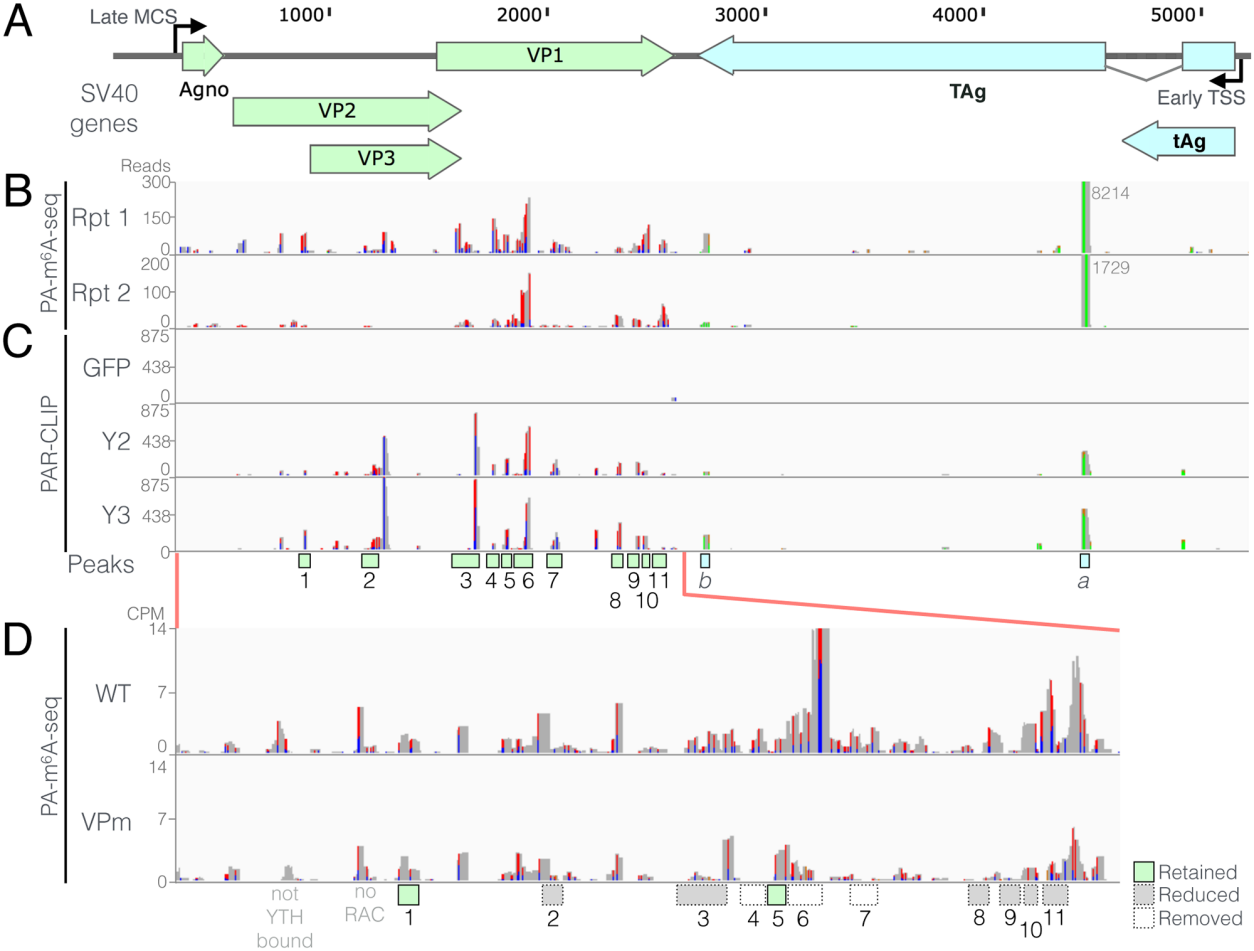
Mapping of m^6^A sites on wildtype and mutant SV40 transcripts. (A) Schematic of the SV40 genome showing coding regions. Nucleotide coordinates are derived from RefSeq NC_001669, SV40 strain 776. MCS: Major late cap site; TSS: early transcription start site. TAg: Large T antigen; tAg: small t antigen. (B) Sites of m^6^A modification on SV40 transcripts were mapped by PA-m^6^A-seq, using SV40- infected BSC40 cells collected 32 hours post-infection (hpi). Two replicates are shown. The numbers next to peak “*a*” in the SV40 early region show the read count for this off-scale peak. (C) SV40 m^6^A sites were also mapped by PAR-CLIP using BSC40 cells expressing FLAG-tagged forms of the m^6^A reader proteins YTHDF2 and YTHDF3, or GFP as a negative control, as described in Fig 1. Infected cells were collected ~36 hpi. 4SU-based CLIP techniques result in T-to-C mutations at cross-linked 4SU residues, that arise during reverse transcription, here shown as red-blue bars. A-to-G mutations, observed on reverse orientation transcripts, are shown as green-orange bars. Candidate m^6^A sites, that were consistently recovered across multiple PA-m^6^A-seq and Y2/Y3 PAR-CLIP experiments, are marked by boxes under Fig. 3C, with early region sites labeled *a* and *b* while the more numerous late region m^6^A sites are numbered 1 through 11. (D) Synonymous mutations were introduced into the 11 mapped late transcript m^6^A sites, disrupting 5’-RRACH-3’ m^6^A motifs, to generate the VPm mutant virus (as S2 Fig). Residual m^6^A sites on VPm were then mapped by PA-m^6^A-seq. The efficiency of removal of each m^6^A site is denoted by the numbered boxes, where white boxes denote undetectable m^6^A levels, gray boxes denote partial m^6^A site removal, while green boxes show no obvious reduction of m^6^A. WT and VPm peak heights are shown normalized to read counts per million host genome assignable reads (CPM).

As shown in Fig 3B, the PA-m^6^A-seq technique gave rise to a reproducible pattern of m^6^A clusters. Only two clusters were detected in the SV40 early region though one of these, towards the 5’ end of the second exon of TAg, was intense. In contrast, we detected ~11 potential m^6^A clusters in the SV40 late region, most of which were present in the VP1 open reading frame (ORF) that also forms the 3’UTR of the viral VP2 and VP3 mRNAs. Because reverse transcription of crosslinked 4SU residues frequently gives rise to a characteristic U>C mutation, we were able to verify that the early and late regions of the SV40 genome were indeed transcribed in opposite orientations (Fig 3A). That is, we observed numerous U>C mutations in the late region of SV40, as shown by red/blue bars, and numerous A>G mutations in the SV40 early region, as shown in orange/green bars (Fig 3B).

Analysis of m^6^A sites using the distinct PAR-CLIP technique showed little or no recovery of SV40 sequences when FLAG-GFP was used as the bait, as expected, while a strong and reproducible pattern of reads, clustering at specific locations in the transcribed regions of the SV40 genome, was achieved when using either FLAG-YTHDF2 or FLAG-YTHDF3 (Fig 3C). While not identical, the m^6^A pattern on SV40 transcripts detected using PAR-CLIP was nevertheless clearly similar to the pattern observed using PA-m^6^A-seq. For example, both techniques identified two identical m^6^A peaks in the SV40 early region and 5 shared peaks towards the 5’ end of the VP1 ORF (Fig 3).

Based on these data, we identified 11 regions within the SV40 late region as potential m^6^A sites. m^6^A addition occurs in a consensus sequence that is minimally 5’-RA*C-3’, where R is purine, but the consensus has been reported to extend somewhat further, as 5’-RRA*CH-3’, where H is anything but G [2,20]. All 20 consensus m^6^A addition sites located within the 11 regions “boxed” in Fig 3C were then mutated, with 16 being mutated in the core 5’-RAC-3’ motif while the other four sites were mutated at the H position, by introduction of a G residue (S3 Fig). The mutations chosen were designed to fully or partially disrupt the 5’-RRACH-3’ consensus without changing the underlying coding potential of the VP1, VP2 and VP3 ORFs (S3 Fig).

To determine if the introduced mutations had indeed reduced m^6^A addition, we used the PA-m^6^A-seq procedure to compare the pattern of m^6^A sites on wild type SV40 with the pattern seen on the m^6^A site mutant shown in S3 Fig, called VPm. As shown in Fig 3D, this analysis revealed that 3 of the 11 m^6^A peaks, including the most intense peak #6, were entirely lost in the VPm mutant virus while six additional m^6^A sites showed reduced recovery. Finally, two m^6^A read clusters, #1 and #5, appeared to not be impacted by the introduced mutations even though neither cluster retained any 5’-RAC-3’ motifs. Therefore, m^6^A is either being added at a non-consensus site or these are not, in fact, sites of m^6^A addition. As expected, mutagenesis of the m^6^A sites in the SV40 late region had no effect on the level of addition of m^6^A to transcripts derived from the SV40 early region (S4 Fig).

**Fig 4:**
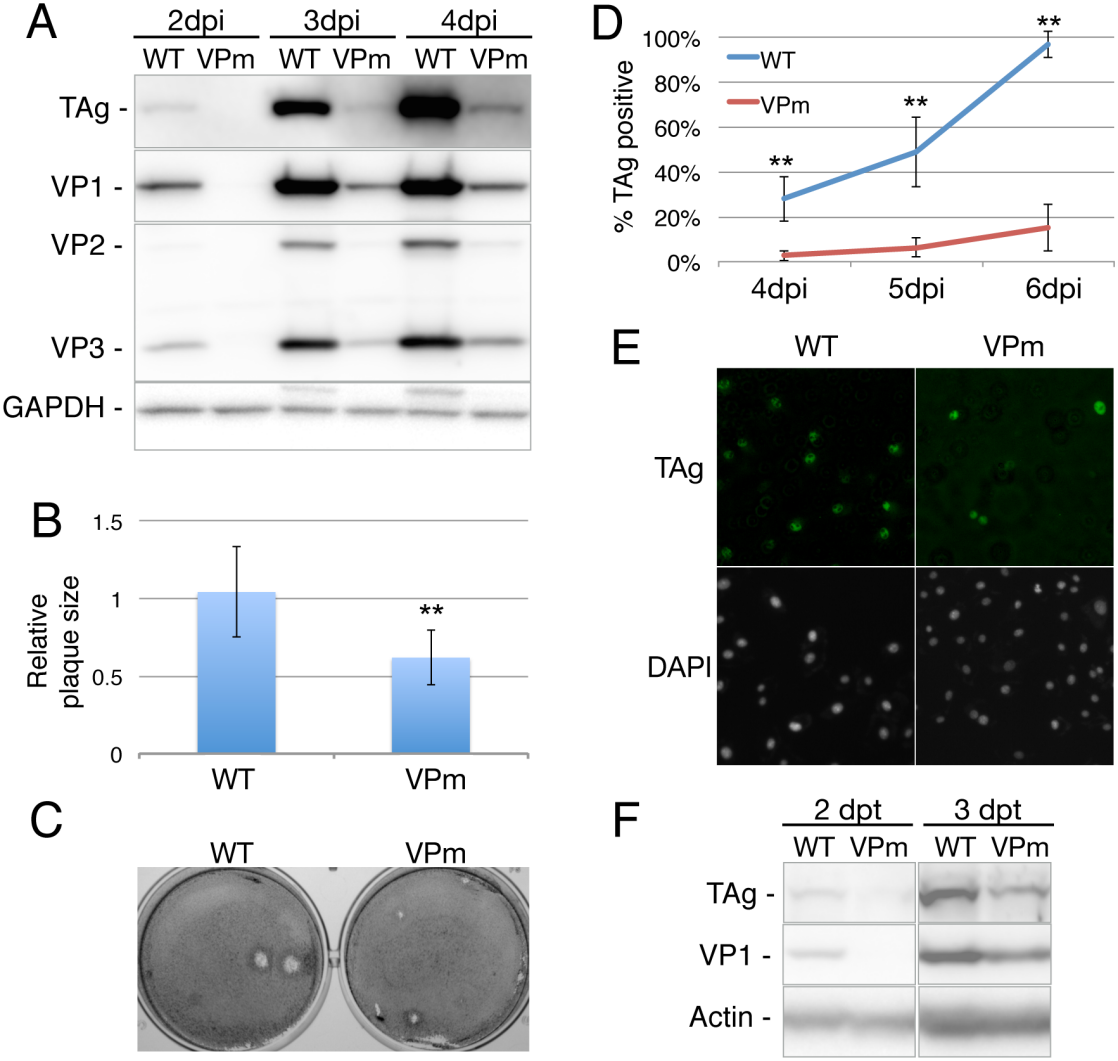
Silent mutational ablation of m^6^A sites reduces SV40 replication. (A) BSC40 cells were infected at an MOI of 0.003 with wild type (WT) or VPm virus, and harvested at 2, 3 or 4 days post infection (dpi). Lysates were then probed for expression of the indicated SV40 proteins by Western blot, with endogenous GAPDH used as a loading control. (B) WT and VPm virus replication was analyzed by quantification of virus plaque size (n = 8) on wild type BSC40 cells, with the average plaque size observed for wild type SV40 set at 1.0. Average of three independent experiments, with SD indicated, using three separate stocks of WT and VPm virus. **p<0.01. (C) Representative photographs of plaques generated by SV40 WT and the VPm mutant. (D) Spread of WT or VPm virus on BSC40 cells was measured by immunofluorescence using an SV40 TAg-specific antibody. (E) Representative immunofluorescence photograph of WT and VPm infected BSC40 cells at 5 dpi, stained with TAg specific antibody and the DAPI nuclear stain. (F) BSC40 cells were transfected with WT or VPm SV40 genomic DNA, and probed for SV40 proteins by Western blot 2 or 3 days post-transfection (dpt).

We next asked if the substantially reduced level of m^6^A addition to SV40 late transcripts documented in Fig 3D would affect SV40 replication in cultured BSC40 cells. We therefore infected wild type BSC40 cells at an MOI of 0.003 with either wild type SV40 or the VPm mutant and analyzed virus spread by Western blot for SV40 proteins at 2, 3 and 4 hpi (Fig 4A) and by immunofluorescence for the SV40 TAg (Fig 4D and 4E) or the SV40 VP1 protein (S5 Fig). As may be readily observed, the VPm mutant replicated significantly (p<0.01) less well than wild type SV40 as measured by any of these distinct assays. To further extend these data we also analyzed the replication potential of the VPm SV40 mutant on two other permissive simian cell lines, CV-1 and Vero. As shown in S6 Fig, we again saw a clear delay in the spread of the VPm mutant, when compared to wildtype SV40, when assayed by Western blot (S6A Fig). In addition, we again observed a significant (p<0.01) ~2-fold reduction in plaque size on CV-1 cells for the VPm mutant when compared to wild type SV40 (S6B and S6C Fig).

We next asked if the VPm mutant would also give rise to smaller plaques, as previously observed in cells lacking the key m^6^A reader protein YTHDF2 (Fig 2). Indeed, we observed that plaques induced by the VPm mutant were ~2-fold smaller in diameter than seen with wild type SV40 (Fig 4B and 4C). Finally, we asked if the VPm mutant would give rise to reduced viral gene expression if introduced into BSC40 cells by transfection, rather than by infection. As shown in Fig 4F, we again observed a reduction in both SV40 TAg and VP1 expression in BSC40 cells transfected with the VPm mutant virus, when compared to wild type SV40.

**Fig 5:**
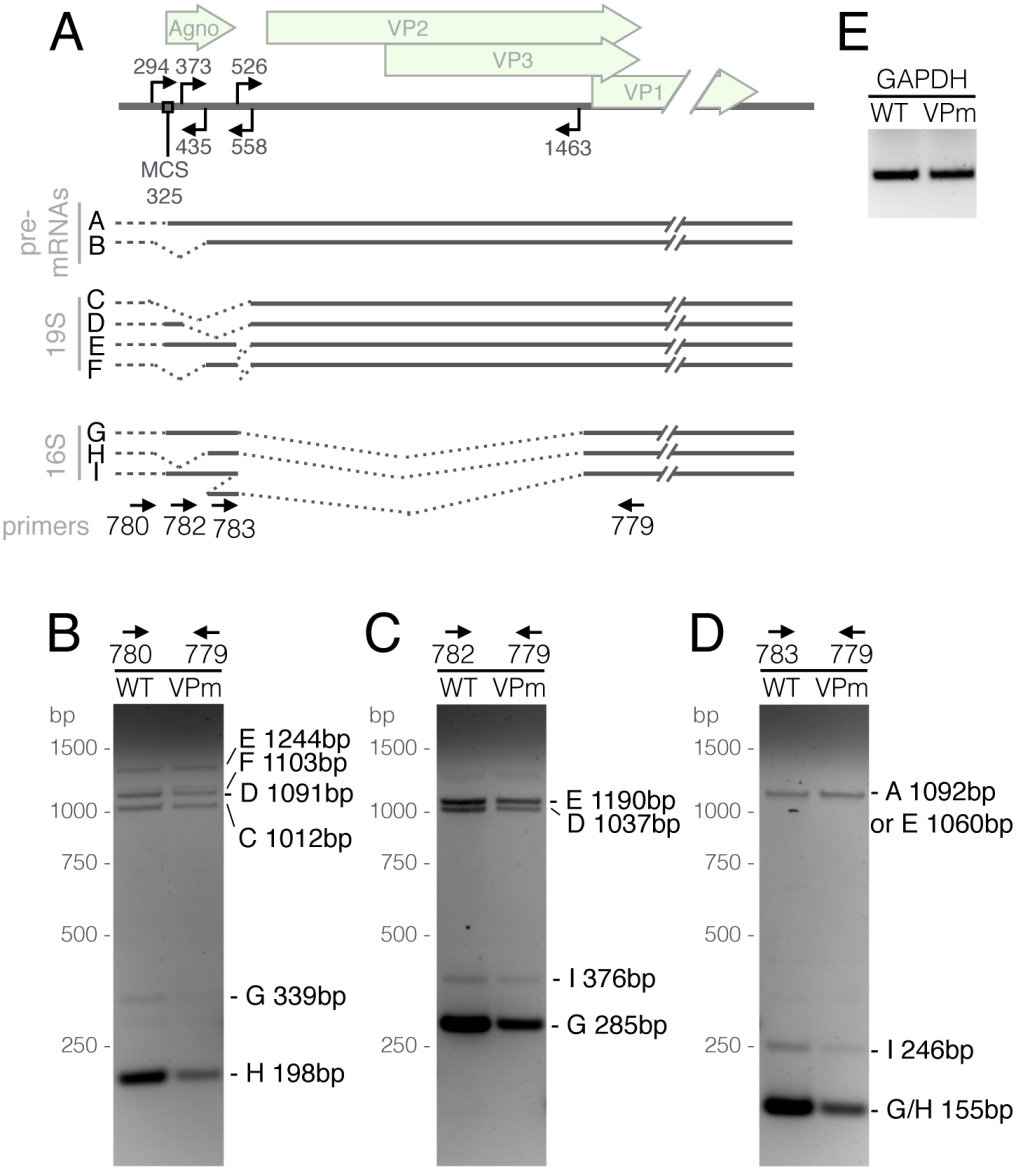
m^6^A does not detectably affect SV40 late transcript alternative splicing. (A) Schematic showing alternatively spliced isoforms of the SV40 late transcripts [45]. Arrows facing in the 3' direction denote 5’ splice sites, while 5'-facing arrows denote 3’ splice sites. Dashed lines at the 5' end of the transcripts represent the known variability of the late transcription start sites. MCS: Major cap site at position 325. Arrows at the bottom of panel A show the different primers used for RT-PCR. Total RNA from WT or VPm-infected cells was reverse transcribed and then subjected to PCR using primer sets 780/779 (B), 782/779 (C), 783/779 (D) Each band is labeled on the right with a letter denoting the spliced isoform it represents, and the expected PCR product size. (E) A GAPDH primer set was used as a loading control.

While the data presented in Figs 1, 2 and 3 argue that the cytoplasmic YTHDF2 protein plays a key role in enhancing the replication of SV40 by interacting with m^6^A present on the SV40 late transcripts, we were also interested in whether the reduced level of m^6^A present on SV40 late transcripts would have any detectable effect on the complex splicing pattern characteristic of these mRNAs, given that the nuclear YTHDC1 protein has been proposed to regulate alternative splicing of cellular mRNAs [5]. For this purpose, we infected wild type BSC40 cells at an MOI of 0.01 with wild type SV40 and the VPm mutant and then allowed these viruses to spread through the culture. At 5 dpi, we harvested total RNA from both infected cultures, reverse transcribed an RNA aliquot using random primers and then subjected the resultant cDNAs to PCR using the four primers described in Fig 5A, which are designed to detect all the various mature viral mRNA isoforms that originate from the SV40 late region (Fig 5A). As shown in Fig 5B, 5C and 5D, while we were able to detect all the predicted late mRNA species in both the wild type SV40 and VPm-infected cells, we did not observe any significant differences in the pattern of isoform expression, although we did consistently detect a lower overall level of all the SV40 late mRNAs in the VPm-infected culture, as expected. We therefore conclude that the mutations introduced into m^6^A-addition sites in the late region of SV40 are not markedly affecting viral mRNA splicing. It remains possible that, if all m^6^A residues could be removed, a more marked effect on viral mRNA splicing might be observed.

**Fig 6:**
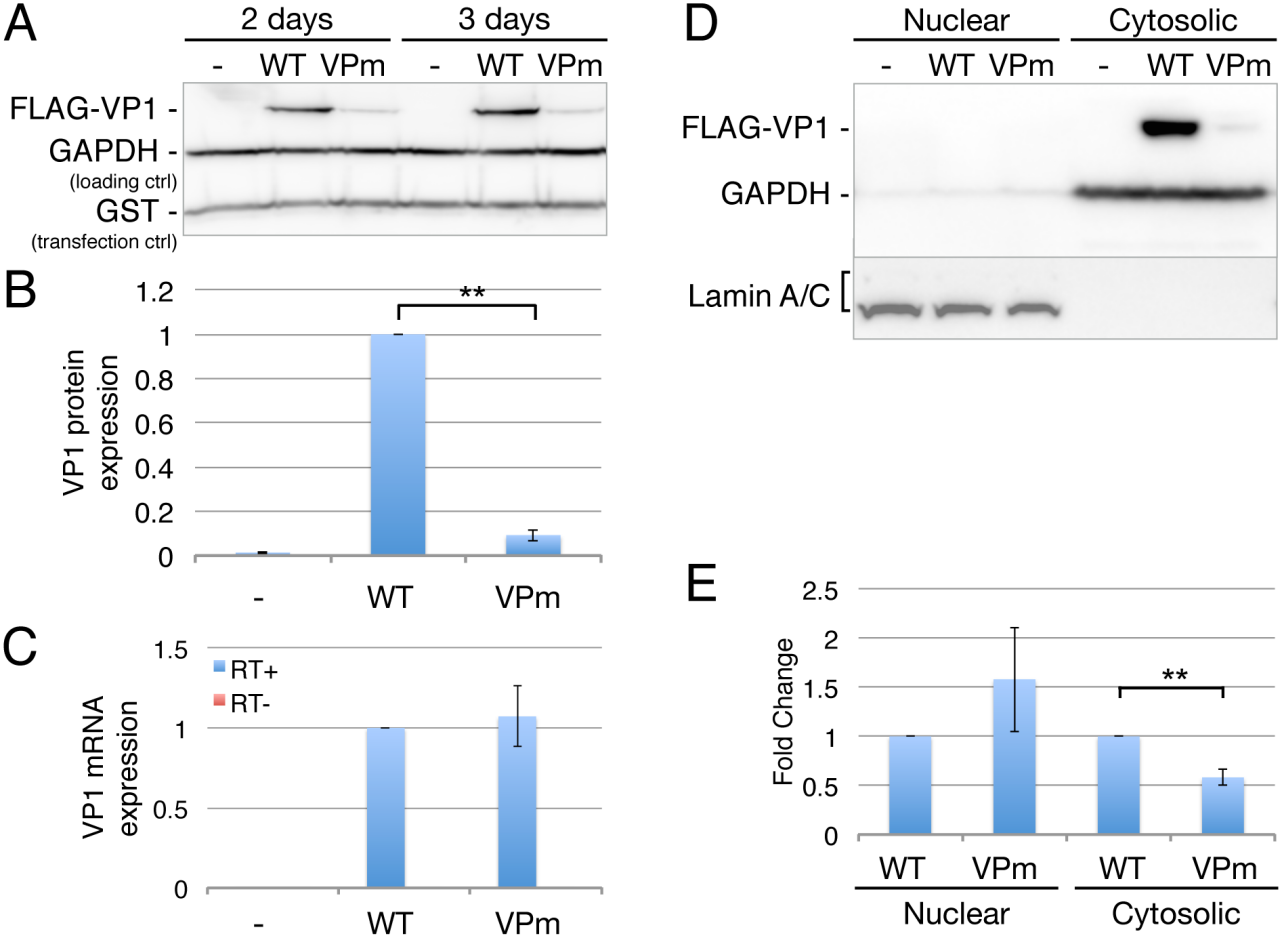
Mutated m^6^A sites in VPm function in the ORF of VP1. (A) The SV40 VP1 gene was cloned from SV40 WT or VPm using PCR and then inserted into an expression plasmid with the addition of a FLAG epitope tag. Transfected 293T cells were probed for VP1 expression by Western blot, with endogenous GAPDH used as a loading control and GST used as a transfection control. (B) The expression level of VP1 from three independent transfection experiments was quantified and normalized to the GST control. The level of VP1 protein detected in cells transfected with the wild type VP1 cDNA were then set at 1.0. (C) VP1 mRNA levels were quantified in parallel using qRT-PCR. No reverse transcriptase (RT-) values shown in red as a negative control. (D) Subcellular fractionation of vector (-), VP1 (WT), or VPm-transfected cells, with Lamin A/C as the nuclear fraction marker and GAPDH as the cytosolic marker. (E) VP1 mRNA levels in the nuclear and cytosolic fractions were quantified in parallel using qRT-PCR. Error bars = SD. **p<0.005 by two-tailed T test.

The experiments using the VPm mutant presented in Figs 4 and 5 examine a spreading SV40 infection in BSC40 cells. To examine the effect of m^6^A residues on SV40 late gene expression in a non-spreading context, we generated expression plasmids containing a FLAG-tagged version of the SV40 VP1 ORF by PCR of this region from wild type SV40 or from the VPm mutant (Fig 3). No viral splice sites were retained in this cDNA expression construct. We then transfected 293T cells with these two VP1 expression plasmids, together with a plasmid expressing glutathione-S-transferase (GST) as a transfection control, and analyzed VP1 and GST expression at 2 or 3 days post-transfection. As may be readily observed, the VP1 ORF from the wild type SV40 genome was expressed ~10-fold higher than the similar VP1 ORF derived from VPm, even though the predicted encoded VP1 protein is identical in both expression plasmids (Fig 6A and 6B). We next measured the level of VP1 mRNA in the transfected cells and, surprisingly, we observed no detectable difference in the level of VP1 mRNA expression. To address whether this effect resulted from nuclear retention of the VP1 mRNA, we performed a fractionation of the transfected 293T cells, as previously described [22], to give nuclear and cytoplasmic cell fractions. The purity of these were confirmed by Western blot using GAPDH as a marker for the cytoplasmic fraction and Lamin A/C as a marker for the nuclear fraction (Fig 6D). We then determined the level of VP1 mRNA in each fraction by qRT-PCR. As may be observed (Fig 6E), we observed a modest increase in the level of VP1 mRNA in the nuclear fraction derived from the VPm transfected cells, when compared to the cells transfected with the wildtype VP1 cDNA, that nevertheless achieved statistical significance (p<0.05). In contrast, there was a significant (p<0.01), ~2-fold decline in the level of VP1 mRNA in the cytoplasm of the VPm-transfected cells, when compared to the wildtype VP1 cDNA (Fig 6E), though this two-fold decline is clearly not sufficient to explain the ~10-fold decrease in VP1 protein expression seen with VPm. Given that the total level of VP1 mRNA is closely similar in both the wildtype and VPm-transfected cells (Fig 6B), the reduction in cytoplasmic VPm mRNA levels seems unlikely to be due to a reduction in its cytoplasmic stability and likely instead reflects the modestly less efficient nuclear export on the VPm mRNA [8]. Thus, these data suggest that the m^6^A sites normally present in the VP1 ORF are increasing VP1 expression primarily by increasing VP1 mRNA translation [23–25].

### Inhibition of m^6^A addition inhibits SV40 replication

**Fig 7:**
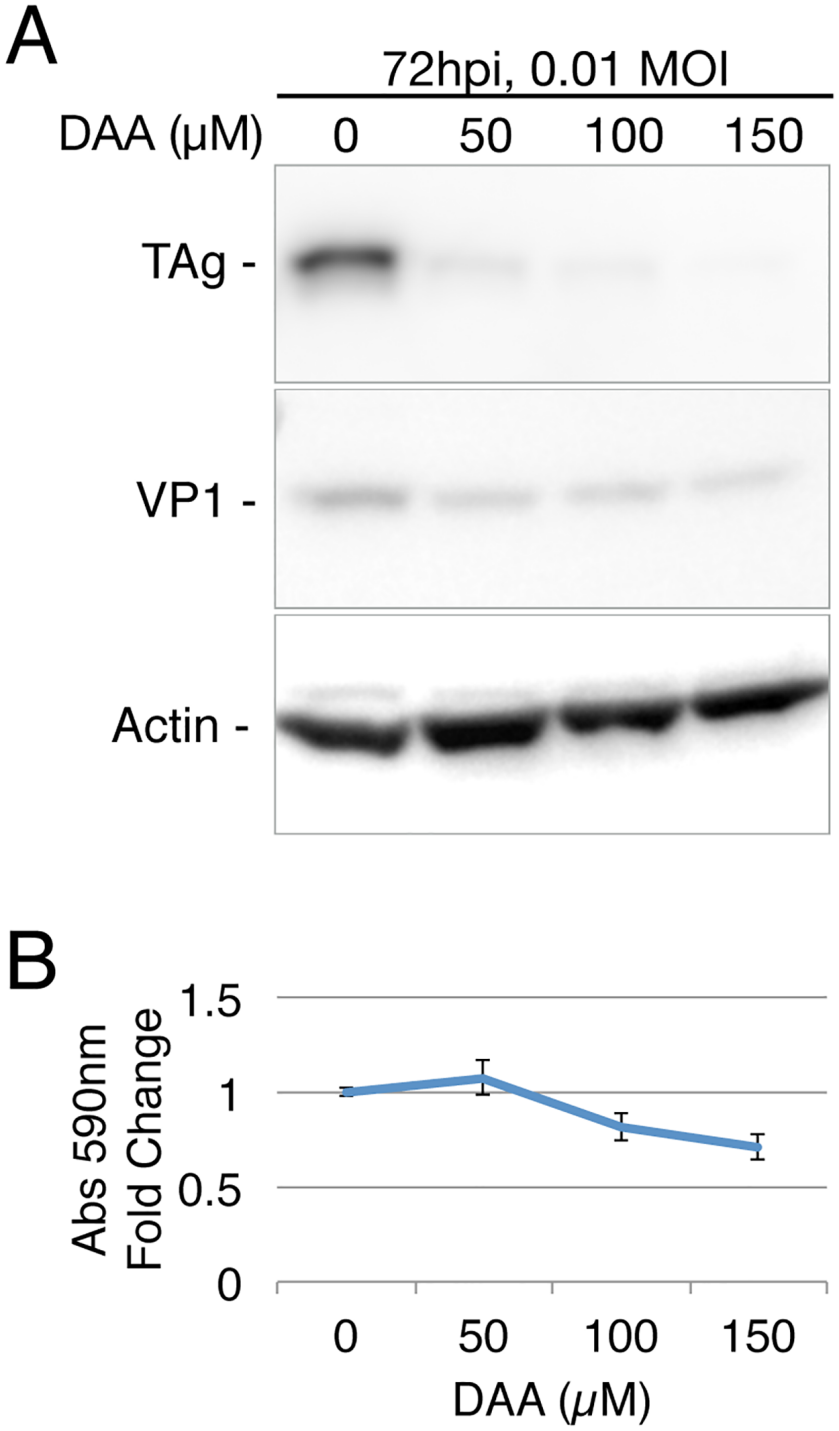
SV40 gene expression is repressed by the methylation inhibitor 3-deazaadenosine (DAA). (A) Western blot of SV40 protein expression in BSC40 cells treated with the indicated concentrations of DAA, infected with SV40 at an MOI of 0.01 and collected at 72 hpi. (B) BSC40 cell viability was analyzed using the MTT assay after 72 hours of DAA treatment.

While no drug able to specifically inhibit METTL3 activity has been described, the drug 3-deazaadenosine (DAA) has been reported to inhibit the addition of m^6^A to mRNA without affecting mRNA capping [26]. DAA acts by inhibiting the formation of S-adenosyl methionine (SAM) from S-adenosyl homocysteine (SAH) and thus is, in principle, a global inhibitor of methylation. However, DAA is in fact well tolerated by cells in culture, and also by animals such as mice and rats, at concentrations that can effectively inhibit m^6^A addition, possibly because METTL3, which uses SAM as its methyl donor, has a weak affinity for SAM and is therefore particularly sensitive to modest reductions in the intracellular SAM concentration [27–30]. When we infected BSC40 cells with wild type SV40 at an MOI of 0.01, and then cultured the cells in various concentrations of DAA for 72 h, we observed a marked reduction in the expression of SV40 proteins, especially TAg, at all concentrations of DAA tested but no decrease in the expression of the cellular protein β-actin (Fig 7A). This latter result is consistent with the observed lack of any significant toxic effect of DAA, especially at the 50 μM concentration, on BSC40 cell viability over the 72-h time course of this experiment as measured by MTT assay (Fig 7B).

## Discussion

The modification of viral mRNAs by addition of multiple m^6^A residues has been reported for a wide variety of both nuclear and cytoplasmic viruses, as well as for several nuclear DNA viruses [11]. The wide prevalence of m^6^A on viral transcripts, and the conservation of sites of m^6^A addition in the genomically plastic lentivirus HIV-1 [13], strongly implies that m^6^A plays some form of positive role in the viral life cycle. Consistent with this hypothesis, we and others have reported that m^6^A addition to HIV-1 transcripts positively regulates HIV-1 gene expression and replication [13,15], and we subsequently extended these results by demonstrating that m^6^A residues not only promote the expression of IAV mRNAs in *cis* but also increase IAV pathogenicity *in vivo* [14]. Recently, it has also been reported that the epitranscriptomic addition of m^6^A residues plays a critical role in the replication cycle of Kaposi’s sarcoma-associated herpesvirus (KSHV) by controlling the splicing, stability and translation of KSHV lytic mRNAs [31]. However, despite the evidence enumerated here arguing that m^6^A positively regulates viral mRNA expression, this issue remains controversial. Specifically, it has been reported that m^6^A reduces the production of HCV virions by infected cells, albeit without affecting viral RNA replication [16]. Moreover, it has also been reported that the distantly related flavivirus ZKV, which expresses viral RNAs that are extensively m^6^A modified, replicates more rapidly when the m^6^A writer METTL3 or m^6^A reader YTHDF2 are knocked down using RNA interference [17], a result which is diametrically opposite to what we have previously reported for IAV [14]. These apparently contradictory results therefore raise the possibility that the interaction of different viruses with the cellular epitranscriptomic machinery can lead to very different outcomes, with m^6^A addition boosting the replication of some viruses, e.g., HIV-1 and IAV, while m^6^A acts to inhibit others, such as ZKV.

Here, we extend our previous work looking at the effect of m^6^A addition on the level of viral gene expression and replication in HIV-1 and IAV [13,14] to an entirely unrelated virus that does not have an RNA genome, the model polyomavirus SV40. As previously also observed for HIV-1 and IAV, we found that overexpression of YTHDF2, a key m^6^A reader, in the SV40-permissive cell line BSC40 substantially increased the level of replication of SV40, as measured by not only Western blot analysis of viral protein expression (Fig 1A) but also viral plaque size (Fig 1C and 1D). Remarkably, we observed a significant increase in not only the size but also the number of SV40 plaques when the same infectious dose of SV40 was analyzed on YTHDF2-overexpressing BSC40 cells (Fig 1B). We do not have a mechanistic explanation for this unexpected result but hypothesize that the YTHDF2 protein is rescuing infections that are otherwise abortive, possibly by boosting viral gene expression early after infection. In any event, the finding that overexpression of YTHDF2 can increase the replication of such widely divergent viruses as SV40, as demonstrated here, and HIV-1 and IAV, as reported previously [13,14], suggests that cell lines overexpressing YTHDF2 might prove invaluable for the growth of a range of attenuated or wild type viruses, destined for use in protective vaccines, by allowing the more rapid production of higher levels of viral particles.

Although the observation that YTHDF2 overexpression can boost SV40 replication (Fig 1), while knockout of *YTHDF2* or *METTL3* in BSC40 cells strongly attenuates SV40 replication (Fig 2), is consistent with the hypothesis that m^6^A addition to SV40 mRNAs is promoting SV40 replication, these effects could clearly be indirect. To address this issue, we mapped the m^6^A residues present on SV40 transcripts using two distinct techniques, PA-m^6^A-seq and PAR-CLIP (Fig 3B and 3C) [20,21], and then introduced a series of silent mutations into the SV40 late region (S3 Fig) that were designed to reduce m^6^A incorporation by altering consensus m^6^A addition sites (5’-RRACH-3’) found coincident with the mapped m^6^A sites. Subsequent analysis revealed that while these mutations indeed reduced m^6^A incorporation into SV40 late transcripts, they did not entirely prevent m^6^A addition (Fig 3D). Nevertheless, we next examined whether these “hypomethylated” late transcripts, expressed by the VPm SV40 mutant, would have any effect on SV40 replication and gene expression. These data revealed that loss of m^6^A sites present in *cis* indeed phenocopied the effect seen upon mutational inactivation of the YTHDF2 gene (Fig 2) in that the VPm mutant not only replicated more slowly than wild type SV40 in BSC40, CV-1 and Vero cells but also produced smaller plaques on both BSC40 and CV-1 cells (Fig 4 and S6 Fig). We therefore conclude that m^6^A addition indeed enhances the expression of SV40 late mRNAs in *cis*.

Given the complex splicing pattern characteristic of SV40 late transcripts (Fig 5A), and given recent data arguing that the nuclear m^6^A reader YTHDC1 regulates the alternative splicing of cellular transcripts [5], we asked whether the reduced level of m^6^A residues present in the VPm mutant might dysregulate late SV40 RNA splicing. Our analysis did not detect such an effect and instead suggested a global reduction in the level of expression of all SV40 late mRNAs (Fig 5B, 5C and 5D). However, when we cloned the VP1 ORF, which contains most of the SV40 m^6^A addition sites (Fig 3), into an expression plasmid, we did observe a strong inhibitory effect on the expression of VP1 as a consequence of the mutational ablation of the m^6^A sites in the VPm mutant (Fig 6A and 6B), even though the encoded VP1 protein is identical in amino acid sequence to wild type VP1 (S3 Fig). Surprisingly, this effect appeared to be primarily at the level of mRNA translation, as no major difference in the level of VP1 mRNA could be observed by qRT-PCR in either the total cell (Fig 6C) or in the nuclear or cytoplasmic RNA fractions (Fig 6E), although the level of the latter was reduced by ~2-fold for the VPm mutant. In fact, the YTHDF2 m^6^A reader protein has been previously proposed to facilitate the translation of mRNAs bearing m^6^A residues [23,32].

As shown in Fig 3, we also detected two sites on m^6^A addition in the early region of the SV40 genome, marked as *a* and *b* in Fig 3C, and the *a* site was actually the most intense site on the SV40 transcriptome, as measured by the number of recovered reads. We also generated SV40 mutants in which these sites were silently mutated to inhibit m^6^A addition and we confirmed that the *a* site was, in fact, entirely lost while the *b* site was essentially unaffected. However, we did not observe any obvious effect on the rate of virus replication when we compared an early region mutant lacking peak *a* to wild type SV40 (S7 Fig). We note that SV40 actually encodes two microRNAs in the viral late region that are antisense to, and downregulate, the SV40 early transcripts [33]. Moreover, mutational inactivation of these microRNAs, which increases SV40 TAg expression, nevertheless was reported to have no clear effect on SV40 replication in culture. Therefore, it would appear that the level of SV40 early gene expression is not rate limiting, at least in cultured cells.

While SV40 is a simian polyomavirus, several polyomaviruses are important human pathogens. These include Merkel cell polyomavirus (MCPyV), which is the etiologic agent of the majority of cases of Merkel cell carcinoma, an aggressive skin cancer [25], BK virus (BKV), which can cause several complications in transplant patients, including hemorrhagic cystitis [34], and JC virus (JCV), which can cause progressive multifocal leukoencephalopathy (PML), a generally fatal disease, in immunodeficient individuals, including individuals receiving injections of antibodies designed to alleviate autoimmune diseases [35]. Therefore, a drug able to globally inhibit polyomavirus replication would be potentially very valuable. While we have not examined the level of m^6^A addition to transcripts encoded by MCPyV, BKV or JCV, we nevertheless hypothesize that these viruses will be similar to SV40, and indeed to a wide range of other unrelated nuclear viruses, in that they will also utilize m^6^A addition to promote viral gene expression in *cis* [11]. A drug that selectively inhibits m^6^A addition might therefore have the potential to serve as a broad spectrum antiviral if the level of toxicity was acceptable. As proof of principle, we therefore asked whether the drug DAA, which induces a global inhibition of m^6^A addition by inhibiting the production of SAM, the methyl donor used by METTL3 [26,28], might also inhibit SV40 replication in culture at doses that are not detectably toxic, and this indeed proved to be the case (Fig 7). DAA has been reported to inhibit a wide variety of viruses not only in culture but also *in vivo*, in mice and in rats [13,14,27–29], which is again consistent with the hypothesis that m^6^A positively affects the replication of many, but perhaps not all, viruses. Nevertheless, these data do suggest that a drug that can selectively inhibit m^6^A addition to mRNAs, such as a METTL3 inhibitor, might prove capable of inhibiting the replication of a wide range of pathogenic human viruses, including pathogenic polyomaviruses, and could prove especially useful for viruses that cause acute infections. Given recent data arguing that the METTL3-mediated addition of m^6^A residues to mRNAs also promotes the uncontrolled growth of acute myeloid leukemia cells [36], such a compound might also prove useful in the treatment of certain types of cancer.

## Materials and methods

### Cell lines

293T cells were obtained from the ATCC (CRL-11268) and were cultured in Dulbecco's Modified Eagle's Medium (DMEM) supplemented with 5% fetal bovine serum (FBS) and 50 μg/ml gentamicin (Gibco). BSC40, CV-1, and Vero cells were grown in DMEM with 10% FBS and 50 μg/ml gentamicin. BSC40 and CV-1 African Green Monkey kidney cells were generous gifts from Dr. Christopher Sullivan of the Department of Molecular Biosciences at the University of Texas in Austin.

BSC40 clones constitutively expressing FLAG-YTHDF2, FLAG-YTHDF3 or FLAG-GFP were produced by transduction using previously described lentiviral expression vectors [13]. YTHDF2 knockout cells, and the corresponding control BSC40 cells, were produced by transduction with LentiCRISPRv2 vectors [37] with sgRNAs targeting exon 4 of the YTHDF2 gene (5'-AGTTACTACAGTCCCTCCAT-3') or GFP (5'-GTAGGTCAGGGTGGTCACGA-3'). Lentiviral vectors were packaged as previously described [14]. Transduced BSC40 cells were selected with 7 μg/ml puromycin for two weeks, then cloned by limiting dilution and maintained in media with 5 μg/ml puromycin. Sequencing of the YTHDF2 CRISPR edited region was done by PCR amplifying a 571nt region containing the edit site from knockout cell line DNA extracts, using the primers oKT0823: 5’-aaaGAATTCCCCAAGTAGACAGGGTTTCG-3’ and oKT0824: 5’-aaaAAGCTT TAGGTGCATAAGCATAATTGCTACTATATCC-3’. The PCR product was EcoRI/HindIII cut and ligated into the pGEM-3zf+ vector (Promega) and transformed into E.coli. Nine individual clones were subject to Sanger sequencing using primer oKT0823. METTL3 knockdown BSC40 cells (ΔM3 cells) were produced using the same protocol using the sgRNA (5’- CTGAAGTGCAGCTTGCGAC-3’), but without limiting dilution cloning.

### Antibodies

The following antibodies were obtained from commercial sources: Large T antigen, clone PAb416 (ThermoFisher, MA1-90661); VP1 (Abcam, ab53977); VP2/3 (Abcam, ab53983); YTHDF2 (Proteintech, 24744-1-AP); METTL3 (Abnova, H00056339-B01P); FLAG antibody clone M2 (Sigma, F1804); GAPDH (Santa Cruz, sc-47724); β-Actin (Santa Cruz, sc-47778; or Invitrogen, MA5-15739); Lamin A/C clone E-1 (Santa Cruz, sc-376248); m^6^A (Synaptic Systems, 202 003); Alexa Fluor 488 goat anti-mouse IgG (Life Technologies, A11029); Alexa Fluor 488 goat anti-rabbit IgG (Life Technologies, A11034).

### Virus preparation

WT SV40 was produced using the SV40 genome plasmid pSV-B3 as described with slight modifications [38]. The SV40 sequence present in pSV-B3, a gift from Dr. Christopher Sullivan (UT Austin), was confirmed by DNA sequencing. The SV40 genome was excised from pSV-B3 by BamHI digestion, followed by gel purification. The purified linear viral genome was then re-circularized using T4 DNA ligase (NEB), and the resultant circular viral genome concentrated using a Microcon 30 concentrator (Amicon). 2 μg of circularized viral DNA was then transfected into 600,000 BSC40 cells in 6-well plates with 4.5 μl of Lipofectamine 2000 (Invitrogen), following the manufacturer’s instructions. Each well of transfected cells was expanded into a T25 flask the next day and cultured in DMEM containing 2% FBS. Virus was collected when cytopathic effects were prominently observed, approximately 8–10 days post-transfection. The entire cell culture in each flask, with media, was then freeze-thawed 3 times, and cell debris then removed by centrifugation. The collected supernatant was aliquoted and frozen at -80°C for storage [39]. Virus stock DNA copy number was determined by qPCR on virus-containing supernatants diluted 10^-5^ in dH_2_O, with primers targeting the TAg encoding region (5'-AACCTATGGAACTGATGAATGG-3' and 5'-TGGAGGAGTAGAATGTTGAGAG-3') [40], alongside a standard curve of serially diluted pSV-B3 DNA. A more accurate titer of each virus stock was then determined as plaque forming units (pfu) by plaque assay on BSC40 cells (as detailed in the next section).

To produce a high copy number virus production plasmid that replicates in E. coli efficiently, the SV40 genome was excised from pSV-B3 by BamHI digestion and ligated into a pGEM-3zf+ (Promega). The resulting pGEM-SV40 plasmid was subsequently sequenced and used for mutant virus generation as well as production of WT virus.

The VPm mutant virus was produced from the pGEM-SV40 plasmid as follows: Peak 11 (as in Fig 3) in the late region was first mutated by PCR of the viral DNA segment from the KpnI site in the pGEM-SV40 vector 5' of the BamHI insertion site, to the KpnI site 5' of the Agnoprotein ORF (coordinate 294 of RefSeq NC_001669), using a 5' PCR primer containing a GAC>GAT mutation (5’- aaGGTACCCGGggatccagaTatgataagatacattg-3’ and 5’- taggtaccttctgaggcggaaagaac-3’). The PCR product was then KpnI digested and ligated back into KpnI digested pGEM-SV40, producing a single nucleotide mutation in the peak 11 region. The SV40 late region between the HaeII (coordinate 836 nt) and BamHI sites (at 2533 nt) was then synthesized as Geneblocks (IDT) with the remaining 5’-RRACH-3’ m^6^A motifs mutated. The core RAC was mutated whenever the encoded amino acid could be preserved; otherwise, nucleotides of the extended 5’-RRACH-3’ motif were mutated. The whole mutant HaeII-BamHI block was PCR amplified from the two outer ends to form a single fragment with HaeII and XbaI ends, then ligated into the HaeII/XbaI sites of pGEM-SV40, generating the pGEM-SV40-VPm plasmid. After confirmation of the predicted sequence by DNA sequencing of the entire cloned region (S3 Fig), virus from pGEM-SV40-VPm was produced by BamHI digestion, re-circularization, and transfection, as described above. Three individual sets of WT & VPm SV40 stocks were separately prepared for use as biological replicates in subsequent infection studies.

Early region m6A mutants were introduced by recombinant PCR of the pGEM-SV40 KpnI-KpnI fragment, then ligated back into the KpnI sites. Primers used include the outside primers oKT0628 5’-tcGGTACCCGGggatccagac-3’, oKT0633 5’-taggtaccttctgaggcggaaagaac-3’, primers to mutate peak 1: oKT0629 5’-gaggagtagaatgCtgagaAtcagcagtag-3’, oKT0630 5’-ctactgctgaTtctcaGcattctactcctc-3’, and primers to mutate peak 2: oKT0631 5’-gtttcaggCtcagggggaggtgtgggaggCtttttaaag-3’, oKT0632 5’-ctttaaaaaGcctcccacacctccccctgaGcctgaaac-3’. Virus production from the mutant viral genome plasmids was done as described above.

### Plaque assays

Plaque assay were performed as described [39], with the following modifications: BSC40 cells were seeded in 6-well plates at 350,000 cells/well, then infected the next day with 200 μl/well of virus stocks at 10^-6^, 10^-7^, and 10^-8^ dilutions in DMEM with 2% FBS. Infection was allowed to proceed for 1 hour, plates gently shaken every 15 minutes. Each well was then supplemented with 1 ml of DMEM with 2% FBS. The media were removed 1 day post-infection (dpi), and infected cells overlaid with 2 ml of DMEM with 5% FBS, 3.7 g/L NaHCO_3_, and 0.8% agarose. Infected cells were fed once more at 4–6 dpi with 1.5 ml of phenol red-free Modified Eagle's Medium (MEM) with 5% FBS, 3.7 g/L NaHCO3, and 0.8% agarose. When plaques could be seen (8–10 dpi), the agarose overlay was removed and the cells fixed and stained with 0.5% crystal violet in 80% methanol and 20% PBS. Plaques were imaged on a Syngene G.box imaging system with each experiment imaged at the same magnification, and plaque sizes measured as diameter in pixels on photos with Image J [41]. SV40 plaque sizes on CV-1 cell monolayers were measured similarly but at 12 dpi.

### Virus infection and transfection for phenotypic studies

BSC40 cells were seeded in 12-well plates at 175,000 cells/well, then infected the next day with 100 μl/well virus stocks, diluted in DMEM with 2% FBS to the desired MOI. Infection was allowed to proceed for 1 hour, plates shaken every 15 minutes. Each well was then supplemented with 0.5 ml of DMEM with 2% FBS. When infections done in a 6-well plate format, the cell number and all media volumes were doubled. Infections of Vero and CV-1 cells were performed similarly except that they were seeded at 150,000 cells/well.

To test the effect of DAA on SV40 replication, BSC40 cells were incubated in DMEM with 2% FBS and various concentrations of DAA (Sigma D8296) for 1 hour prior to infection. The DAA-containing media were then removed and set aside, and SV40 was added to cells at an MOI of 0.01. After 1 hour of infection with occasional shaking, the previously set aside DAA-containing media was added back to the infected cells.

Transfection of viral genomes for phenotypic studies was done as for virus production. Transfected cells were harvested at 2 and 3 days post-transfection for Western blot analysis.

### Viability assay on DAA-treated cells

BSC40 cells were seeded in 96-well plates at 22,000 cells/well in DMEM with 2% FBS. Once cells were attached, the media were replaced with 100 μl/well of media containing various concentrations of DAA. After three days of cell culture in DAA, an MTT assay was performed as described (http://www.abcam.com/kits/mtt-assay-protocol). Briefly, the culture media were replaced with 100 μl/well of 2.5 mg/ml MTT [3-(4, 5-dimethylthiazolyl-2)-2, 5-diphenyltetrazolium bromide] in 0.5X serum-free DMEM and left in the incubator for 4 hours. The resultant MTT product was then dissolved by directly adding 150 μl of 4 mM HCl and 0.1% NP40 in isopropanol, then left on a rotating shaker for 15 mins. The 590 nm absorbance was read with a BMG Labtech CLARIOstar plate reader at the Duke Functional Genomics core.

### Alternative SV40 mRNA splicing assay

BSC40 cells infected with WT or VPm virus at an MOI of 0.01 were harvested at 5 days post-infection and subject to RNA extraction with Trizol (Invitrogen) following the manufacturer’s instructions. 1μg of RNA from each sample was treated with RQ1 DNase (Promega) for 30 mins, then reverse transcribed to cDNA using Super Script III with random hexamers (Invitrogen). The cDNA was then subject to PCR with GoTaq polymerase (Promega) with the forward primers 780 (5’-agctggttctttccgcctc-3’), 782 (5’-atttcaggccatggtgctgc-3’), 783 (5’-gaaggggaagatactgttgacggg-3’); and the reverse primer 779 (5’-gcccctggacaacttccttttc-3’). The GAPDH control primers were 5'-tgggtgtgaaccatgagaag-3' and 5'-gatggcatggactgtggtc-3'. PCR products were visualized on a 1.5% agarose gel.

### m^6^A mapping

For PA-m^6^A-seq, 3.5 million cells/flask of BSC40 cells were seeded in nine T75 flasks, then infected the next day with 7.7x10^4^ viral DNA copies per cell of SV40 in 3 ml/flask of DMEM with 2% FBS. For mutation validation PA-m^6^A-seq, BSC40 cells were seeded in ten 15 cm plates at 4.8 million cells/plate, then infected the next day at an MOI of 3.0 in 6 ml of DMEM/2%FBS per plate. After one hour of infection, with occasional shaking, cells were topped up with 8 ml media, supplemented with 4SU to a final concentration of 100 μM. Infections were harvested 36–40 hpi, and total RNA extracted with Trizol (Invitrogen). The mRNA fraction was purified with the Ambion Poly(A)Purist MAG kit (ThermoFisher, AM1922). 10 μg of poly(A) RNA was then subject to pulldown and cross linking with 7.5 μg of m^6^A antibody (Synaptic Systems 202–003), following the published protocol [20].

PAR-CLIP was done as previously described [21]. BSC40 cells constitutively expressing FLAG-tagged GFP, YTHDF2 or YTHDF3 were each seeded in eighteen 15 cm plates at 8.3 million cells/plate, then infected with SV40 at an MOI of ~10 in 4 ml DMEM with 2% FBS. After one hour of infection, with occasional shaking, cells were topped up with 11 ml of media. At 2 hpi, infected cells were supplemented with 1 ml additional DMEM with 4SU to a final concentration of 100 μM. At 38 hpi, infected cells were irradiated twice with 2500×100 μJ/cm^2^ of 365 nm UV, harvested and the PAR-CLIP protocol performed using a mouse monoclonal anti-FLAG antibody (Sigma F1804, clone M2), as previously described.

TruSeq library preparation and Illumina sequencing were done as previously described [14]. Sequencing reads longer than 15 nt with fastq quality scores above 33 were used for analysis. Reads were aligned to the SV40 genome (RefSeq NC_001669.1) using Bowtie [42], allowing up to 1 mismatch. An in-house Perl script was then used to screen for 4SU crosslink-induced T>C mutations, where reads that do not fit the following criteria were discarded: reads aligning to the forward strand of the viral genome need to contain a T>C mutation; reads aligning to the reverse strand of SV40 need to contain an A>G mutations. All data were processed with SAMtools and visualized with IGV, as previously described [13,43,44]. For the validation of VPm removal of m^6^A, peak heights were normalized to the total host assignable read count of each sequencing run. Reads longer than 15 nt with fastq quality scores above 33 were aligned with Bowtie to the African Green Monkey genome (Chlorocebus_sabeus 1.1/chlSab2, Vervet Genomics Consortium GCA_000409795.2) downloaded from the UCSC genome browser, allowing up to one mismatch, then filtered with the same T>C mutation script. T>C-mutation containing host assignable read counts for the WT and VPm PA-m^6^A-seq runs were 48,166,426 and 55,603,658, respectively. Early region m^6^A mutant validation peaks were normalized to total high quality reads (>15nt, fastq quality score >33, contains index sequence) to calculate counts per million (CPM).

### Immunofluorescence microscopy

BSC40 cells in 6-well plates were infected at an MOI of 0.003. Cells were reseeded the next day onto glass cover slips in 12-well plates, then harvested and fixed at 4, 5, and 6 days post-infection. Fixation and antibody staining were done as described [14], using the T antigen antibody at 1/1000, VP-1 antibody at 1/1000, Alexa Fluor 488 anti-mouse at 1/1000, and Alexa Fluor 488 anti-rabbit at 1/670 dilution.

### VP1 expression vector

The FLAG tagged YTHDF2 cDNA was cloned out of pLEX-FLAG-YTHDF2 [13] into the HindIII/EcoRI sites of the pK-Myc vector, replacing the Myc tag. The SV40 VP1 gene was PCR cloned from either WT or VPm virus infected BSC40 cell cDNA, then cloned into the NotI (between the N'-FLAG and insert gene) and Cla I site (3' of the stop codon) of pK, replacing YTHDF2. pK-FLAG-VP1, and the m^6^A mutant pK-FLAG-VPm, along with the negative control pK-Myc vector, were co-transfected with a GST expression plasmid (pK-GST) into 293T cells with polyethylenimine (PEI) in 12-well plates. Transfected cells were harvested 2 and 3 days post-infection for Western blot or RNA analysis by qRT-PCR. RNA was extracted with Trizol (Invitrogen), treated with RQ1 DNase (Promega), then reverse transcribed to cDNA with the ABI High-Capacity cDNA Reverse Transcription Kit (Thermo Fisher 4368814). Half of the DNase-treated RNA was "reversed transcribed" in the absence of reverse transcriptase and used as the RT- control. qPCR was done with the VP1 primers 5'-taagatggccccaacaaaaa-3' and 5'-tccttttatgacgagctttgg-3' and GAPDH primers 5'-tgggtgtgaaccatgagaag-3' and 5'-gatggcatggactgtggtc-3'. VP1 primers were specifically designed to avoid any m^6^A mutations.

### Subcellular fractionation

293T cells were seeded at 400,000 cells/well in 6-well plates, then transfected with pK-FLAG-VP1 or pK-FLAG-VPm. Each well of cells were passaged into a 10 cm plate the next day, and harvested with trypsin 3 days post transfection. Cells were washed twice in cold PBS, and incubated in NP40 lysis buffer (10 mM Tris-HCl pH 7.5, 10 mM NaCl, 3 mM MgCl_2_, 0.5% _(w/v)_ NP40) [22]. Cell pellets resuspended in 300 μl lysis buffer were vortexed for 5 secs, then left on ice for 5 mins. Nuclei were then spun down in a microfuge at full speed for 10 secs. The supernatant was collected as the cytosolic fraction. The nuclear pellet was washed in another 300 μl of lysis buffer, split into two tubes, and spun down again. Each nuclear pellet and cytosolic fraction were then divided in half for Western blot analysis or RNA extraction with Trizol or Trizol LS (Invitrogen).

## Supporting information

S1 Fig Analysis of YTHDF2 and YTHDF3 overexpressing BSC40 cells. **(A)** Western blot analysis of wild type BSC40 cells and of clones transduced with a lentiviral vector expressing FLAG-GFP (G), FLAG-YTHDF2 (Y2) or FLAG-YTHDF3 (Y3) using a commercial YTHDF2-specific antiserum. Endogenous YTHDF2, and the slightly larger epitope tagged form of YTHDF2, are both detected in the Y2 cells. The similar but larger YTHDF3 protein is detected in the overexpressing Y3 subclone, but not in the parental BSC40 cell line, due to cross-reactivity with the YTHDF2 antiserum. (B) The protein sequence of YTHDF2 from humans, African green monkeys, macaques, and mice was aligned, showing the high conservation in mammals, with 100% protein sequence conservation across these three primates. (C) Similar to panel B except comparing the sequence of the equally highly conserved YTHDF3 protein.
(TIF)

S2 Fig Mutational inactivation of the *YTHDF2* gene. CRISPR/Cas was used to introduce inactivating mutations into the *YTHDF2* gene in BSC40 cells. The sgRNA sequence and the relevant protospacer adjacent motif (PAM) are indicated. Sequencing of 9 independent cDNA clones identified 4 clones with the indicated 41bp deletion and 5 clones with the indicated 143bp deletion, both of which introduce frame shift mutations into the YTHDF2 open reading frame. No wildtype sequence was observed.
(TIF)

S3 Fig Silent mutations introduced into the late region of the VPm virus. DNA sequence alignment of the coding region of VP2/3 and VP1 (562–2593 nt) of WT (strain 776) and VPm SV40, with the encoded amino acid sequence annotated underneath. m^6^A peaks shown in Fig 3 are here shaded in gray, with peak numbers and mutation knockdown efficiency color coded at right (as in Fig 3). Mutated 5’-RRACH-3’ motifs are shown shaded in orange or green. # indicates mutations that disrupt these m^6^A motifs. Preferably, the R, A or C in the core motif triplet was mutated whenever they were found in a codon wobble position (shown in orange), while mutations at the termini of the broader 5’-RRACH-3’ motif were made when the core RAC could not be changed without altering the encoded amino acid (shown in green).
(TIF)

S4 Fig Mutations introduced into SV40 late region m^6^A sites do not affect m^6^A sites on SV40 early transcripts. (A) Schematic of the SV40 genome showing coding regions (see Fig 3A). (B) PA-m^6^A-seq of WT and VPm viral transcripts expressed from the early region (as Fig 3D)
(TIF)

S5 Fig Slower spread of the m6A-mutant virus VPm as assayed by immunofluorescence for the VP1 protein. This experiment was performed as described in Fig 4D and 4E except that the BSC40 cells infected with WT or VPm virus were stained with a VP1 antibody at 5 dpi. (A) Representative photographs of two biological replicates each of WT and VPm-infected cells. (B) Quantification of VP1 expressing cells from three biological replicates each of WT and VPm-infected cells. Error Bars = SD, **p<0.01 by 2-tailed Student's T-test.
(TIF)

S6 Fig Infection of alternative simian cell lines by the SV40 VPm mutant. (A) CV-1 and Vero cells were infected with WT SV40 or the VPm mutant, as described in Fig 4A, and then probed for SV40 protein expression by Western blot. As may be observed, both the SV40 WT and VPm mutant infections spread more slowly in CV-1 and especially Vero cells than seen in BSC40 cells in Fig 4A. (B) Quantification of the size of plaques induced by wild type SV40 and the VPm virus mutant on CV-1 cells, as described in Fig 4B. Physical aberrations at the well edges were not counted. n = 26, **p<0.01. (C) Representative photographs of plaques generated by SV40 wild type and the VPm mutant on CV-1 cells (wells of 10^-6^ diluted virus).
(TIF)

S7 Fig Lack of a phenotype when SV40 early region m^6^A sites were mutated. (A) Schematic of the genetic organization of the SV40 genome. (B) Both peak *a* and peak *b* coincided with two 5’-RAC-3’ motifs. This panel shows PA-m^6^A-seq tracks for the early region of SV40 for the wild type virus, for an early region mutant, Tm1, in which both 5’-RAC-3’ motifs in m^6^A peak *a* were mutated, an early region mutant, Tm2, in which both 5’RAC-3’ motifs in peak *b* were mutated and a third mutant, Tm12, in which 5’-RAC-3’ motifs in both peaks were mutated. As may be observed, peak *a* was totally ablated in Tm1 and Tm12 while peak *b* was not affected by the introduced mutations. Peak heights are shown normalized to read counts per reads (CPM). (C) Because the mutations introduced into peak *b* had no effect on m^6^A addition at this site, we focused our phenotypic analysis on mutant Tm1. This representative Western blot shows that the level of TAg expression in infected BSC40 cells was not detectably affected by loss of m^6^A peak *a*. Experiment performed as described in Fig 4. (D) Same as panel C except this bar graph shows a compilation of data drawn from three independent viral preparation sets for TAg, with SD indicated.
(TIF)
