# Supplementary figures and images for: Addition of m6A to SV40 late mRNAs enhances viral structural gene expression and replication
Source: PLoS Pathog. 2018 Feb 15;14(2):e1006919. doi: 10.1371/journal.ppat.1006919 (PMC5831754; doi:10.1371/journal.ppat.1006919)

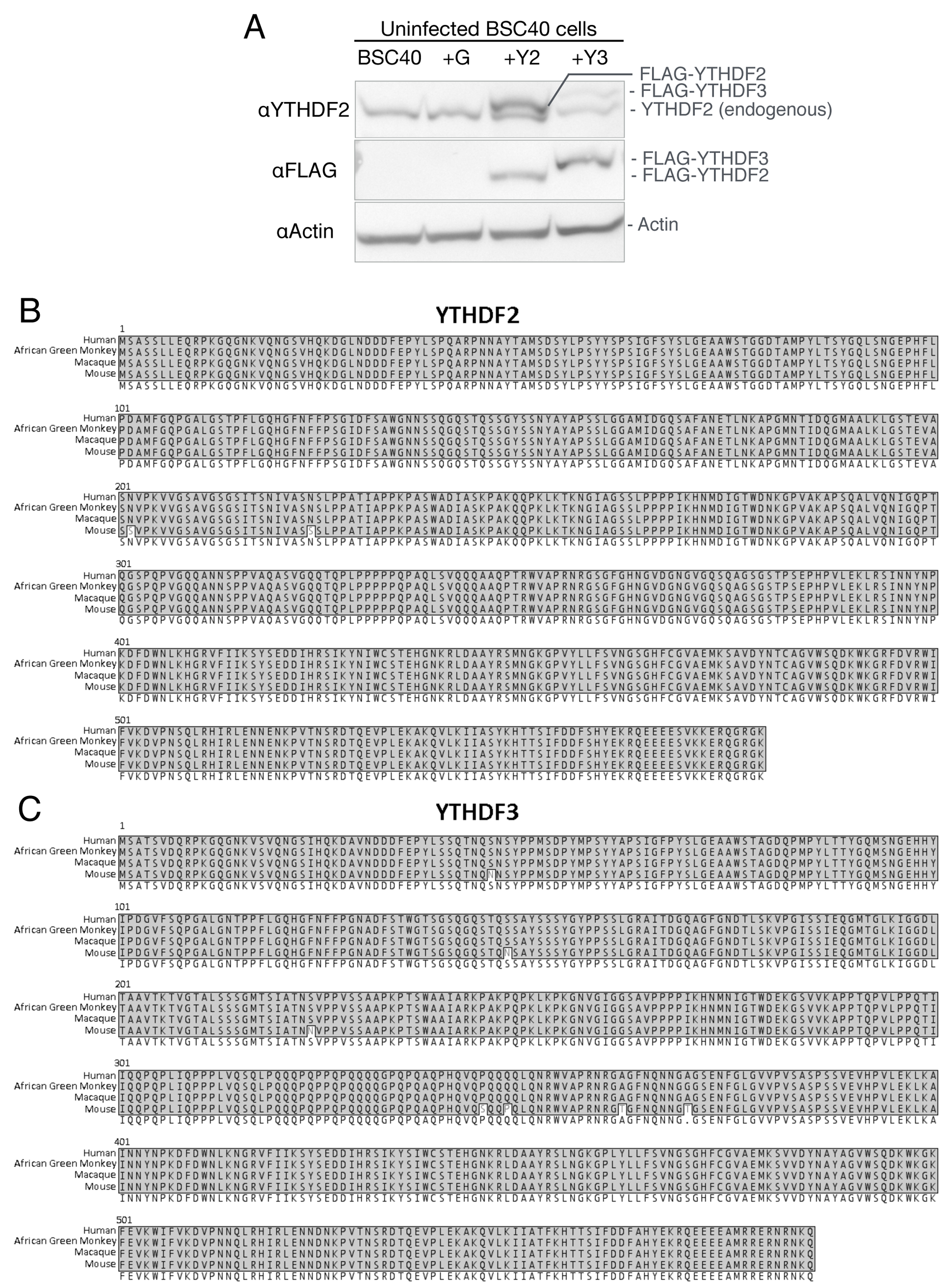

Supplement: S1 Fig — (A) Western blot analysis of wild type BSC40 cells and of clones transduced with a lentiviral vector expressing FLAG-GFP (G), FLAG-YTHDF2 (Y2) or FLAG-YTHDF3 (Y3) using a commercial YTHDF2-specific antiserum. Endogenous YTHDF2, and the slightly larger epitope tagged form of YTHDF2, are both detected in the Y2 cells. The similar but larger YTHDF3 protein is detected in the overexpressing Y3 subclone, but not in the parental BSC40 cell line, due to cross-reactivity with the YTHDF2 antiserum. (B) The protein sequence of YTHDF2 from humans, African green monkeys, macaques, and mice was aligned, showing the high conservation in mammals, with 100% protein sequence conservation across these three primates. (C) Similar to panel B except comparing the sequence of the equally highly conserved YTHDF3 protein. (TIF) [file ppat.1006919.s001.tif]

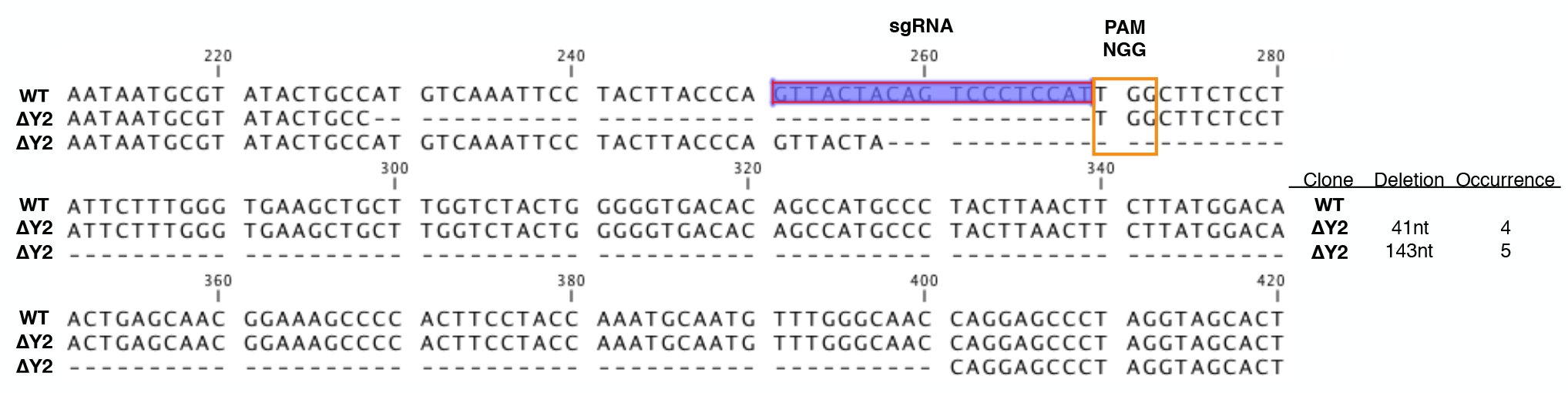

Supplement: S2 Fig — CRISPR/Cas was used to introduce inactivating mutations into the YTHDF2 gene in BSC40 cells. The sgRNA sequence and the relevant protospacer adjacent motif (PAM) are indicated. Sequencing of 9 independent cDNA clones identified 4 clones with the indicated 41bp deletion and 5 clones with the indicated 143bp deletion, both of which introduce frame shift mutations into the YTHDF2 open reading frame. No wildtype sequence was observed. (TIF) [file ppat.1006919.s002.tif]

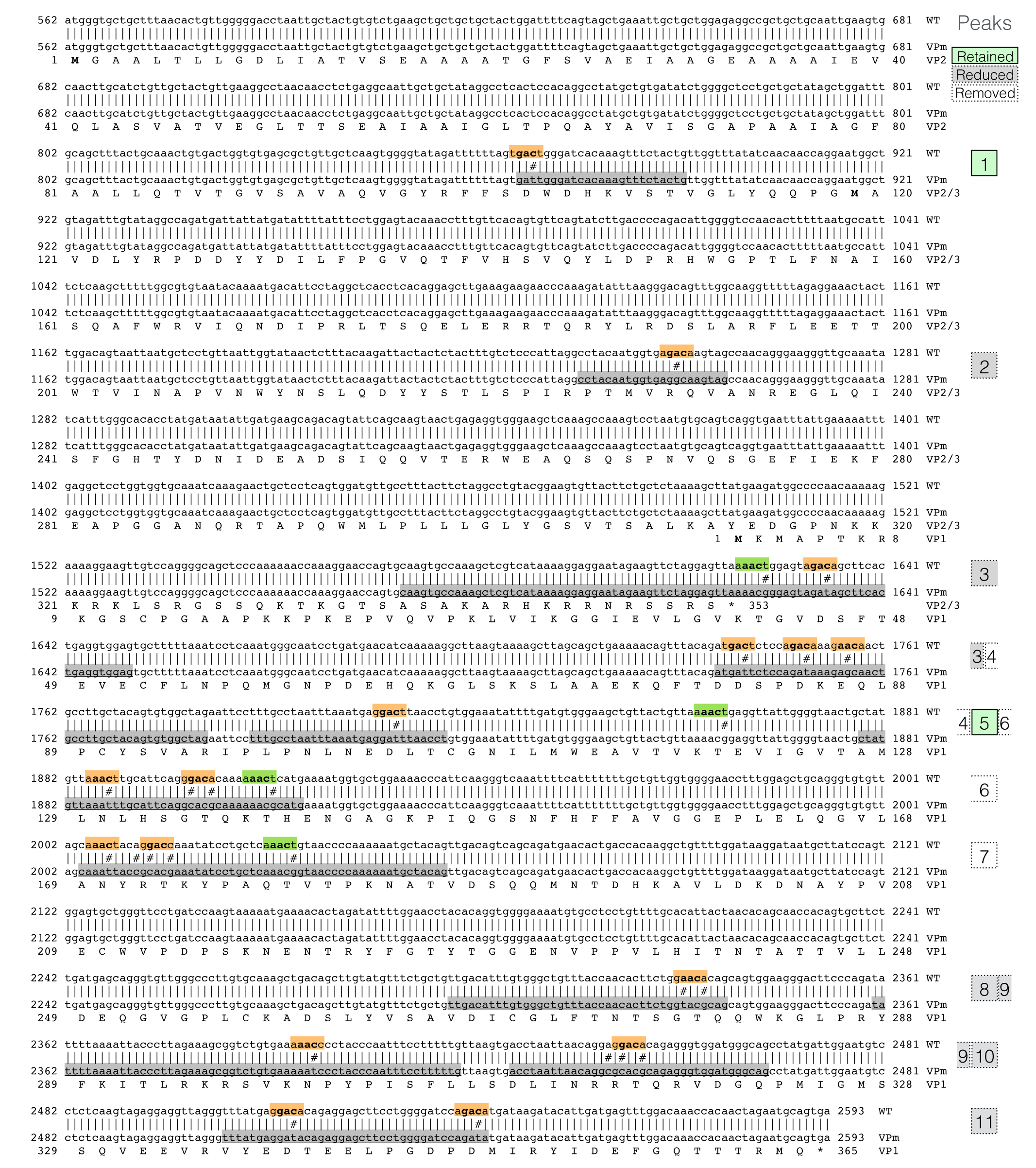

Supplement: S3 Fig — DNA sequence alignment of the coding region of VP2/3 and VP1 (562–2593 nt) of WT (strain 776) and VPm SV40, with the encoded amino acid sequence annotated underneath. m6A peaks shown in Fig 3 are here shaded in gray, with peak numbers and mutation knockdown efficiency color coded at right (as in Fig 3). Mutated 5’-RRACH-3’ motifs are shown shaded in orange or green. # indicates mutations that disrupt these m6A motifs. Preferably, the R, A or C in the core motif triplet was mutated whenever they were found in a codon wobble position (shown in orange), while mutations at the termini of the broader 5’-RRACH-3’ motif were made when the core RAC could not be changed without altering the encoded amino acid (shown in green). (TIF) [file ppat.1006919.s003.tif]

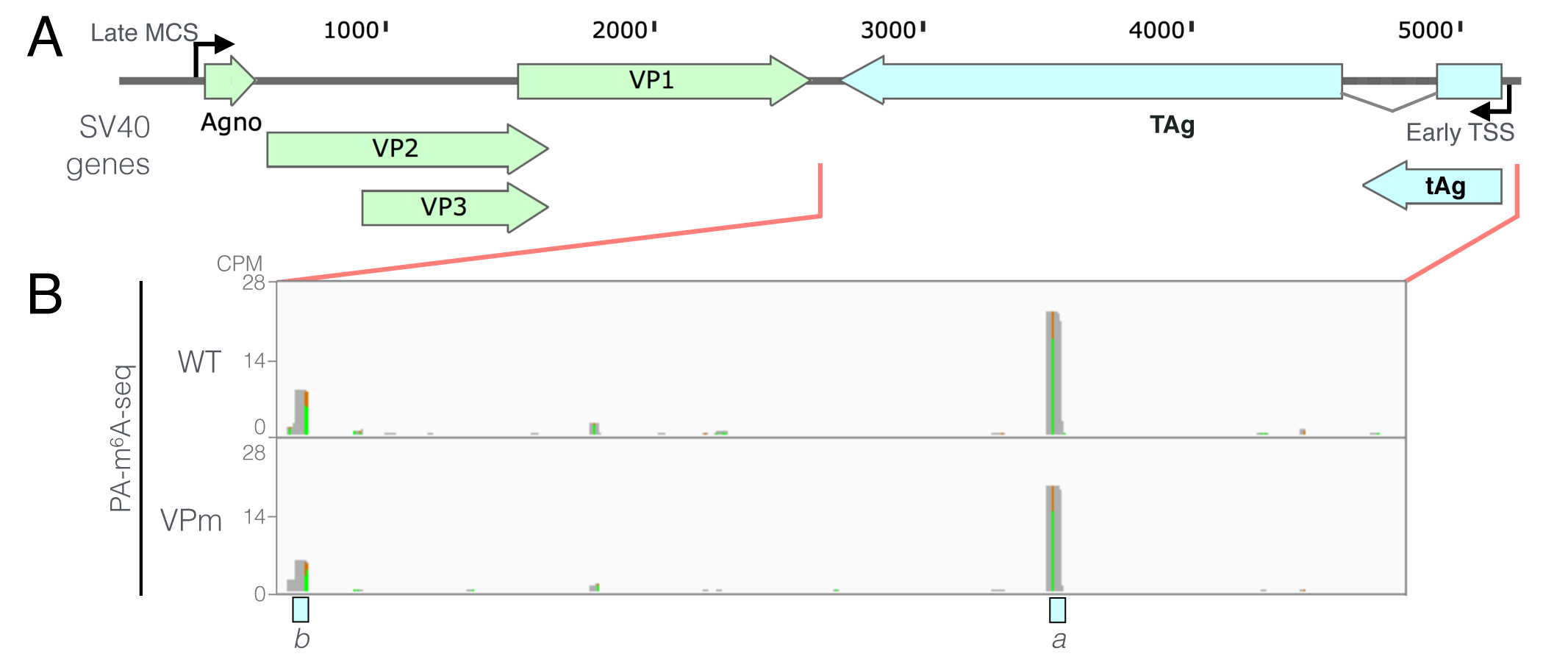

Supplement: S4 Fig — (A) Schematic of the SV40 genome showing coding regions (see Fig 3A). (B) PA-m6A-seq of WT and VPm viral transcripts expressed from the early region (as Fig 3D) (TIF) [file ppat.1006919.s004.tif]

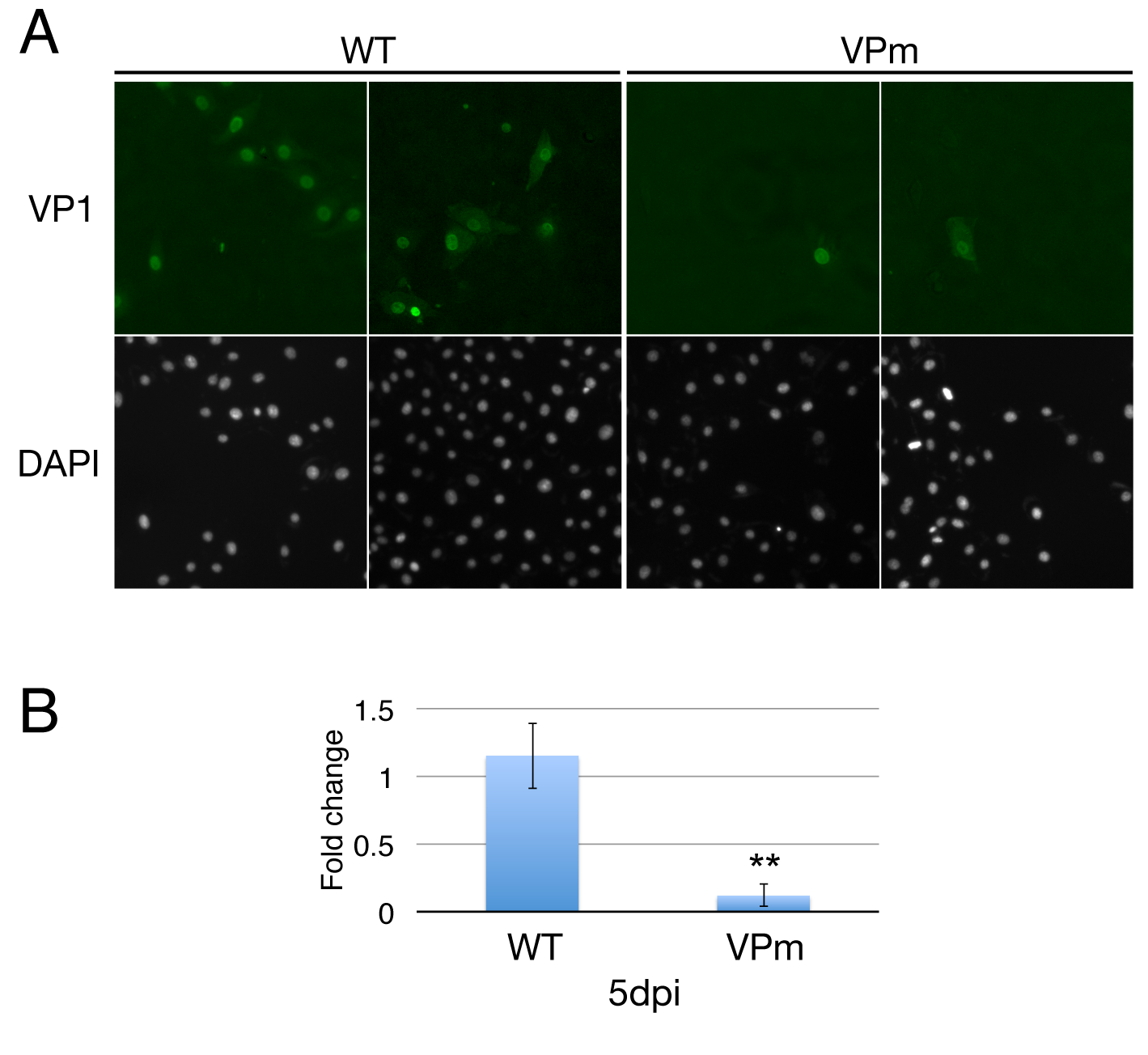

Supplement: S5 Fig — This experiment was performed as described in Fig 4D and 4E except that the BSC40 cells infected with WT or VPm virus were stained with a VP1 antibody at 5 dpi. (A) Representative photographs of two biological replicates each of WT and VPm-infected cells. (B) Quantification of VP1 expressing cells from three biological replicates each of WT and VPm-infected cells. Error Bars = SD, **p<0.01 by 2-tailed Student's T-test. (TIF) [file ppat.1006919.s005.tif]

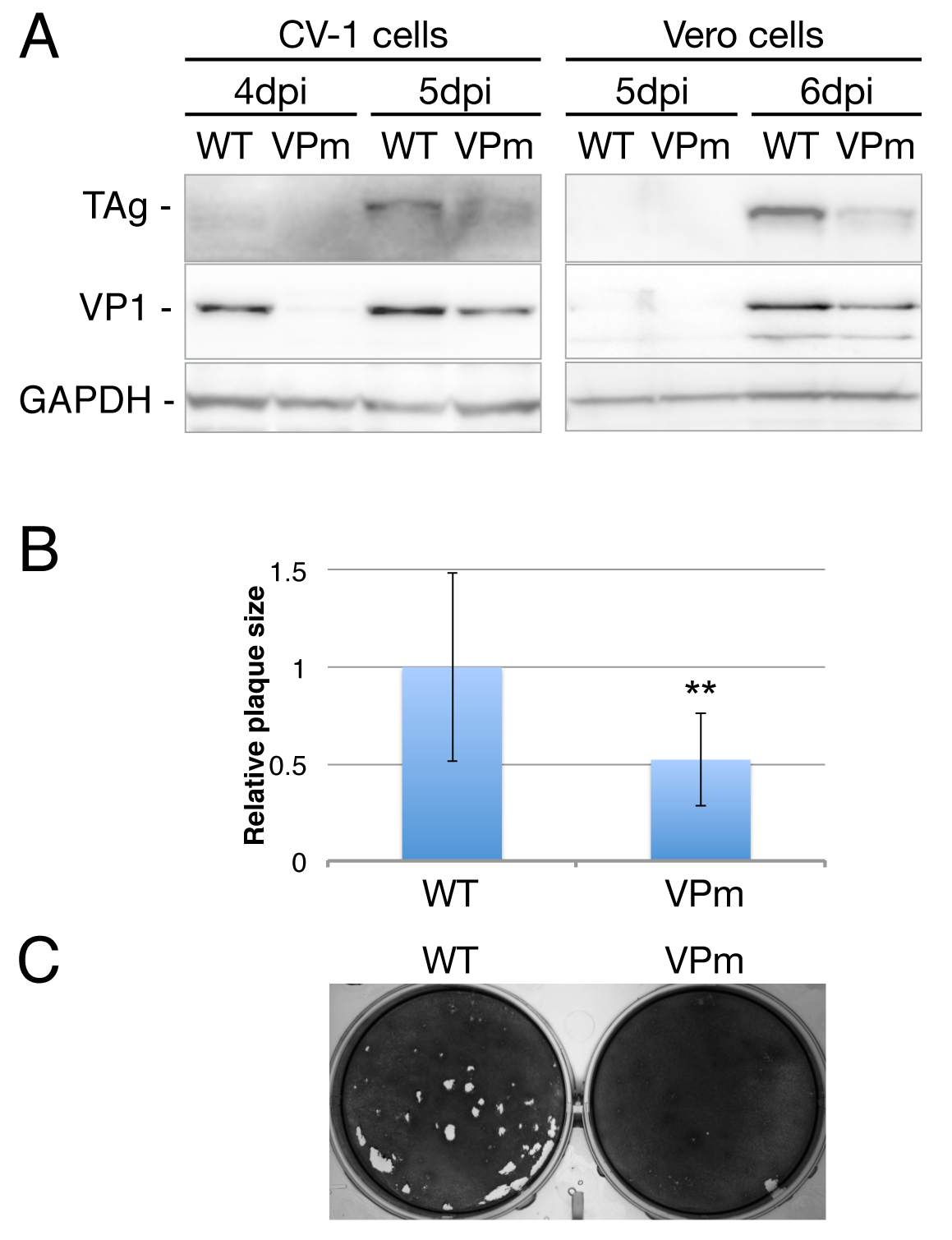

Supplement: S6 Fig — (A) CV-1 and Vero cells were infected with WT SV40 or the VPm mutant, as described in Fig 4A, and then probed for SV40 protein expression by Western blot. As may be observed, both the SV40 WT and VPm mutant infections spread more slowly in CV-1 and especially Vero cells than seen in BSC40 cells in Fig 4A. (B) Quantification of the size of plaques induced by wild type SV40 and the VPm virus mutant on CV-1 cells, as described in Fig 4B. Physical aberrations at the well edges were not counted. n = 26, **p<0.01. (C) Representative photographs of plaques generated by SV40 wild type and the VPm mutant on CV-1 cells (wells of 10-6 diluted virus). (TIF) [file ppat.1006919.s006.tif]

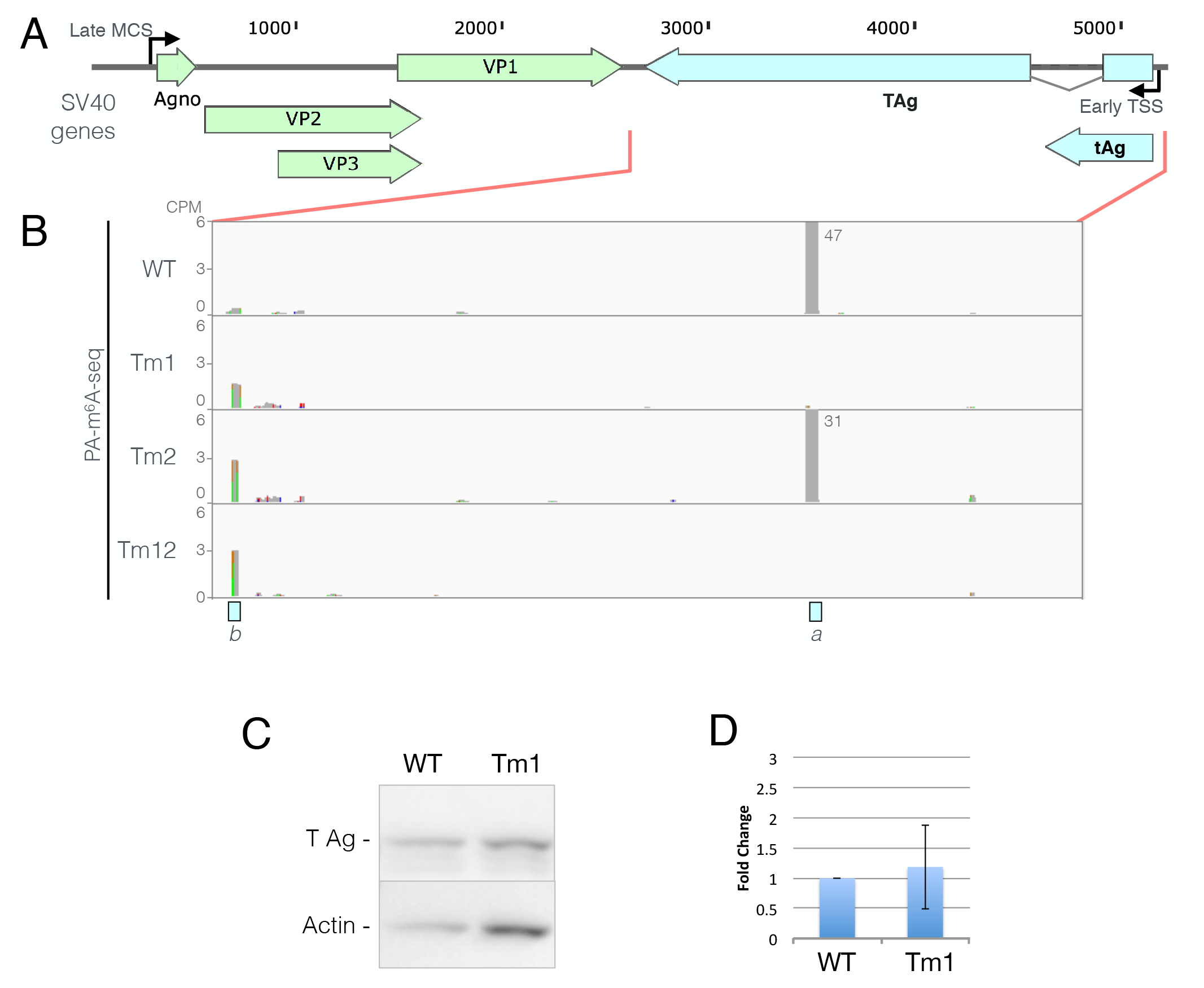

Supplement: S7 Fig — (A) Schematic of the genetic organization of the SV40 genome. (B) Both peak a and peak b coincided with two 5’-RAC-3’ motifs. This panel shows PA-m6A-seq tracks for the early region of SV40 for the wild type virus, for an early region mutant, Tm1, in which both 5’-RAC-3’ motifs in m6A peak a were mutated, an early region mutant, Tm2, in which both 5’RAC-3’ motifs in peak b were mutated and a third mutant, Tm12, in which 5’-RAC-3’ motifs in both peaks were mutated. As may be observed, peak a was totally ablated in Tm1 and Tm12 while peak b was not affected by the introduced mutations. Peak heights are shown normalized to read counts per reads (CPM). (C) Because the mutations introduced into peak b had no effect on m6A addition at this site, we focused our phenotypic analysis on mutant Tm1. This representative Western blot shows that the level of TAg expression in infected BSC40 cells was not detectably affected by loss of m6A peak a. Experiment performed as described in Fig 4. (D) Same as panel C except this bar graph shows a compilation of data drawn from three independent viral preparation sets for TAg, with SD indicated. (TIF) [file ppat.1006919.s007.tif]
